# Supplementary figures and images for: Dynamin independent endocytosis is an alternative cell entry mechanism for multiple animal viruses
Source: PLoS Pathog. 2024 Nov 14;20(11):e1012690. doi: 10.1371/journal.ppat.1012690 (PMC11594517; doi:10.1371/journal.ppat.1012690)

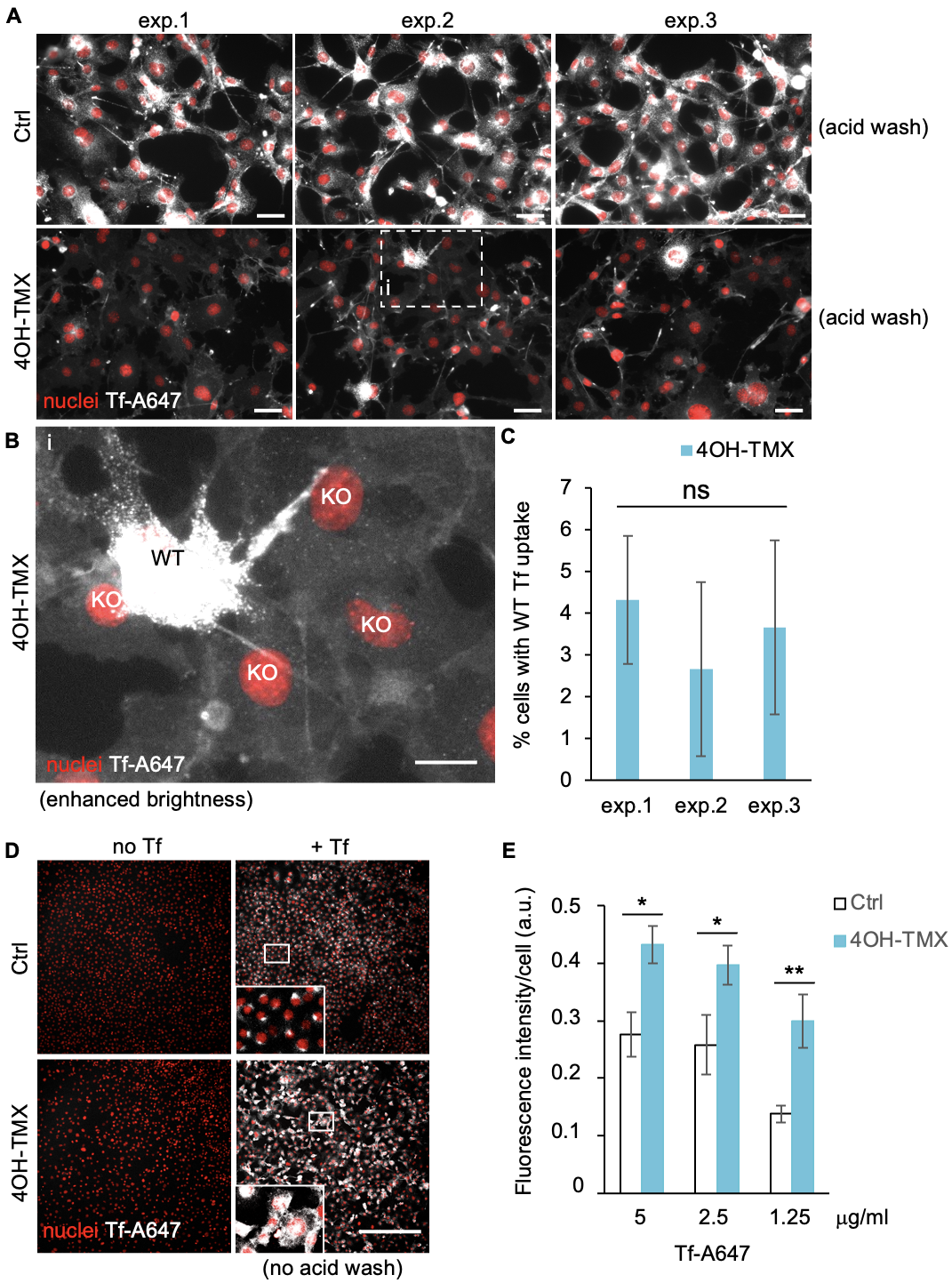

Supplement: S1 Fig — A) Representative fluorescence images of three independent experiments where 20 min uptake of 5 μg/ml Tf-A647 at 37°C was monitored in MEFDKO cells pre-treated with EtOH vehicle control (Ctrl) or 4OH-TMX for 6 days. Before fixation, cells where washed once with growth media at pH 5.5 followed by media at pH 7.4 to remove non internalized Tf from the cell surface (acid wash). Scale bar = 5 μm, nuclei stained with Hoechst (red). B) The white boxed area from panel A was expanded to distinguish cells where the uptake of Tf-A647 was blocked (KO; i.e. the 4OH-TMX-induced KO of dynamins was efficient) from cells that did not respond to 4OH-TMC and internalized levels of Tf-A647 comparable to those of vehicle Ctrl treated cells (indicated as WT in the image). Scale bar = 10 μm, nuclei stained with Hoechst (red). C) Quantification of Tf-A647 uptake shown in A using automated image analysis. A threshold of Tf-A647 fluorescence intensity in the perinuclear area was set to distinguish wild type from KO cells. Values represent the mean of three replicas. Error bars represent the STDEV. Statistical analysis was performed using an unpaired double tailed t-test (ns = non significant). D) Tf uptake was performed as described in A except cells were not acid washed before fixation. Scale bar 100 μm. E) Quantification of Tf-A647 fluorescence per cell after image analysis (a.u. = arbitrary units). Values represent the mean and standard deviation of three independent experiments. Statistical analysis was performed using ordinary two-way ANOVA multiple comparisons test (* p<0.05; ** p<0.01). (TIF) [file ppat.1012690.s001.tif]

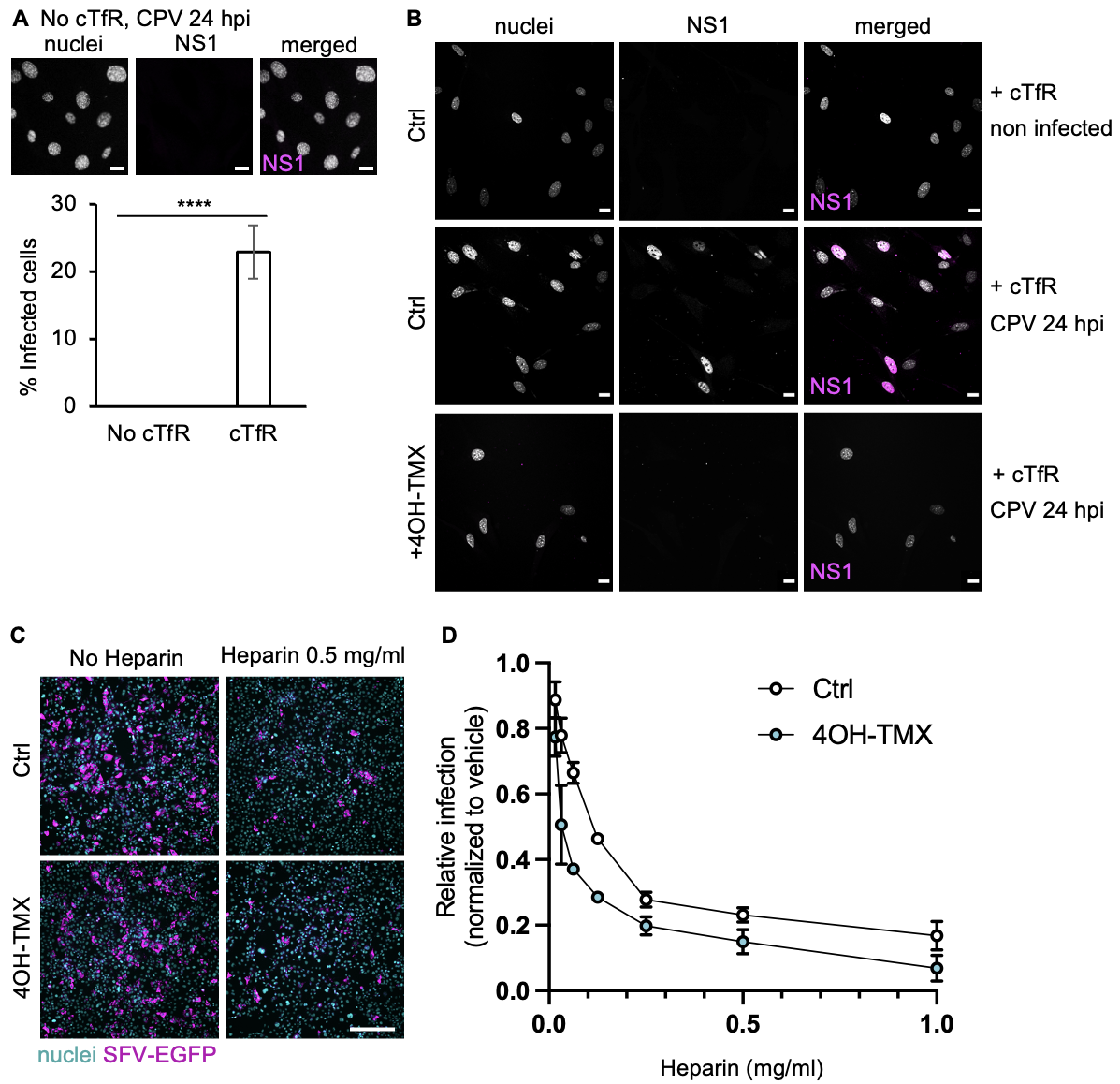

Supplement: S2 Fig — A) Representative confocal fluorescence images of MEFDKO cells infected with CPV for 24 h. Infected cells are visualized by immunofluorescence using an antibody against the viral non-structural protein NS1. Nuclei are visualized with DAPI DNA stain. Scale bar = 5 μm. The % of infected cells quantified after automated image analysis is shown in the graph. Statistical analysis was performed using a two tailed T-test (**** p<0.0001). B) Representative confocal fluorescence images of MEFDKO cells transiently overexpressing the feline TfR and infected with CPV for 24 h after 6 days treatment with EtOH vehicle control (Ctrl) or 4OH-TMX to induce dynamin depletion. Infected cells are visualized by immunofluorescence using an antibody against the viral non-structural protein NS1. Nuclei are visualized with DAPI DNA stain. Scale bar = 5 μm. C) Representative fluorescence image of MEFDKO 6 days after treatment with EtOH vehicle control (Ctrl) or 4OH-TMX and treated with PBS control (no Heparin) or increasing concentrations of Heparin and subsequentelly infected for 7 hours with SFV-EGFP (magenta). Nuclei are stained with Hoechst DNA dye (cyan). Scale bar 100 μm. D) Quantification of the experiments shown in C by automated image analysis. Values represent the mean and standard deviation of the mean of three independent experiments. Values are normalized to the % of infected cells obtained in control (Ctr) and vehicle samples (i.e. no heparin, indicated as 1). (TIF) [file ppat.1012690.s002.tif]

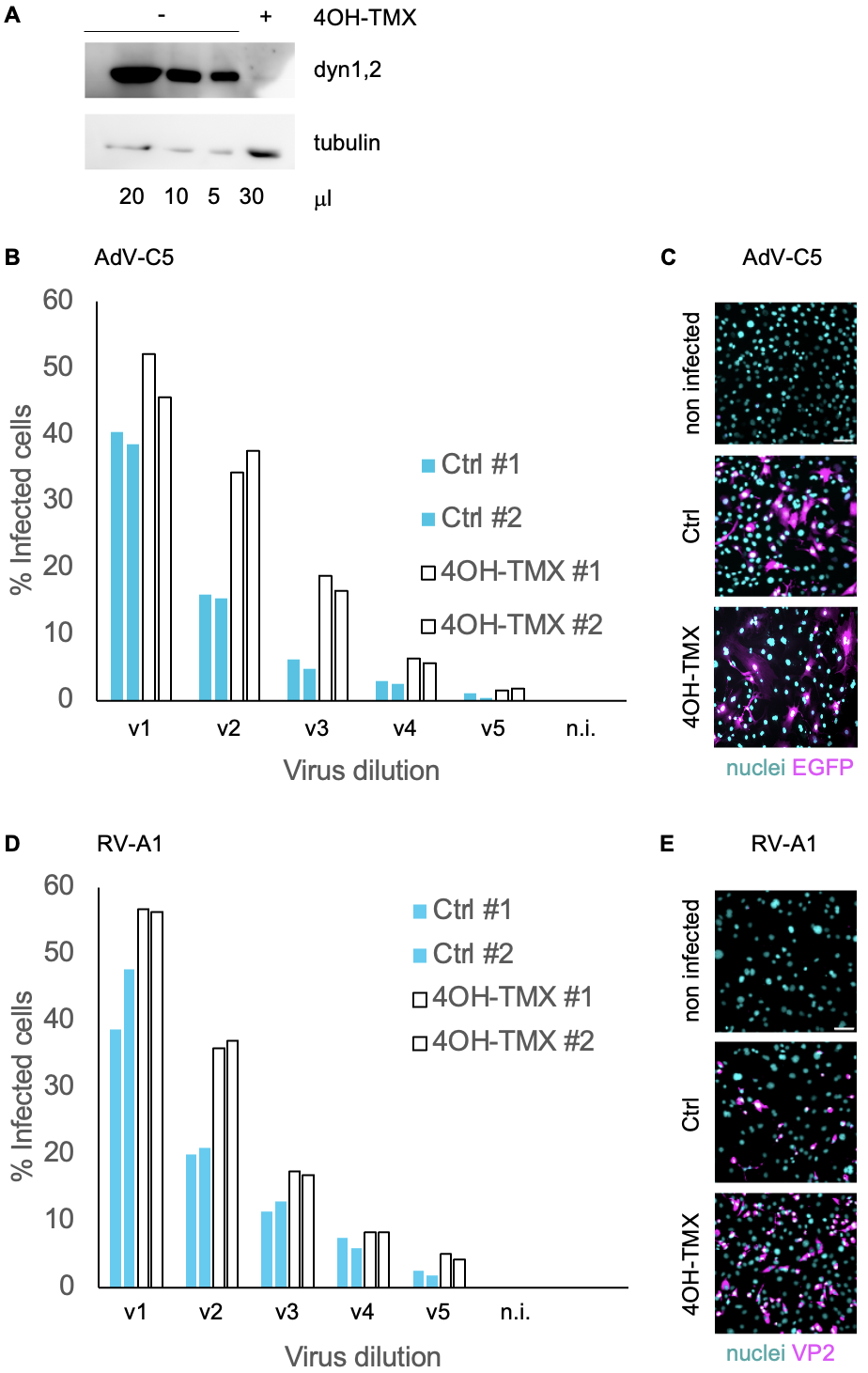

Supplement: S3 Fig — A) Western blot analysis of dynamin 1,2 levels in MEF DNM1,2, DKO cells treated with vehicle control or 40H-TMX for 6 days. Indicated amounts of samples were loaded in the gel before blotting. Tubulin was used as a loading control. The Dyn1,2 antibody used recognizes both dynamin 1 and 2. B-C) Quantification of infection in MEF DNM1,2, DKO cells treated with vehicle control (Ctrl) or 4OH-TMX for 6 days and infected with AdV-C5 for 22 h, and D-E) RV-A1 for 24 h. Virus infection was determined by direct fluorescence imaging of AdV-C5 induced EGFP (C, magenta), and after immunofluorescence staining of RV-A1 VP2 protein (E, magenta). Representative epifluorescence images are shown in C and E. The experiment was performed with two technical replicates (#1 and #2). V1-v5 refer to two-fold dilutions of input virus, n.i. = non infected. Scale bar = 10μm. (TIF) [file ppat.1012690.s003.tif]

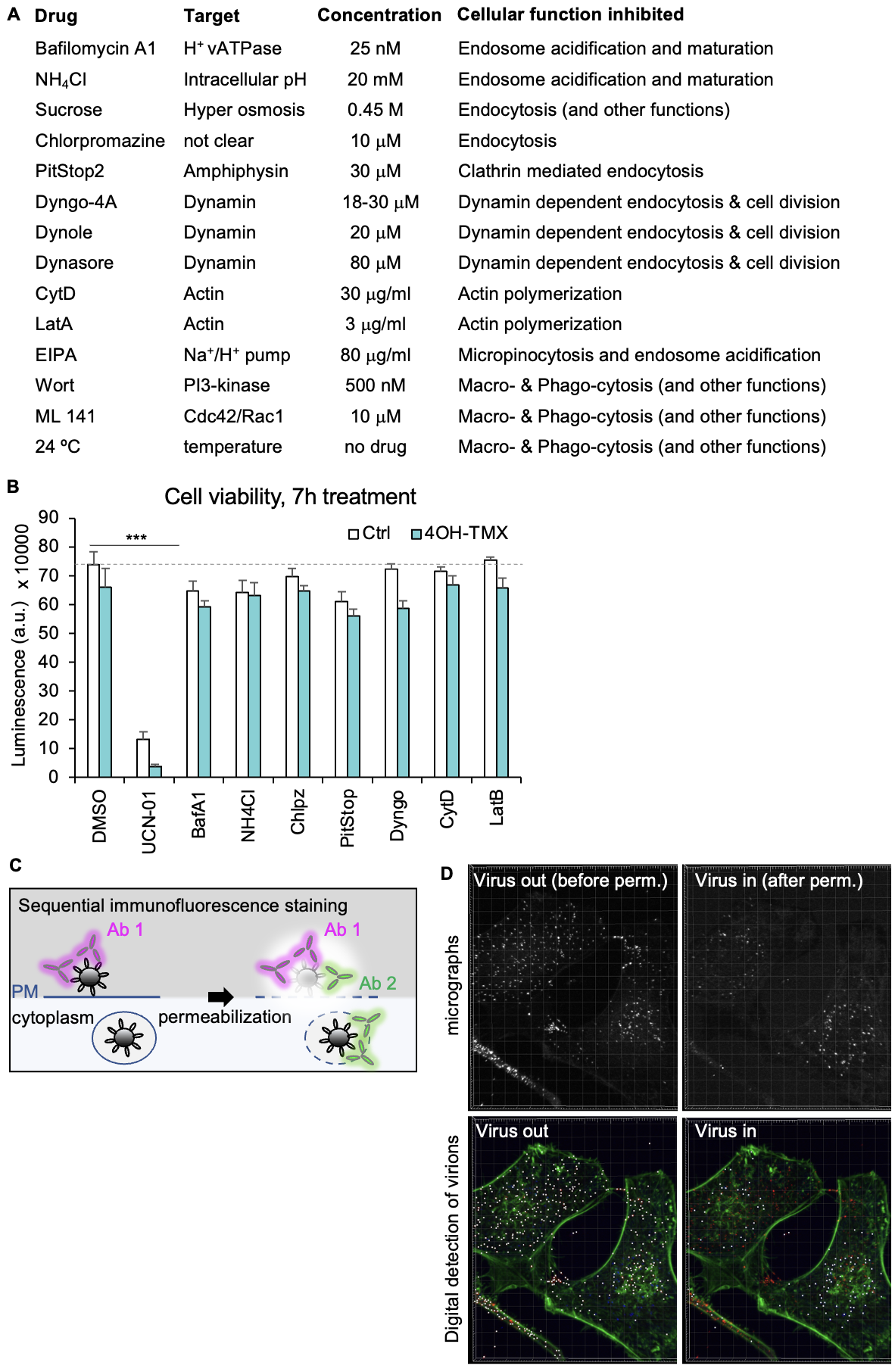

Supplement: S4 Fig — A) List of the drugs used for SFV experiment shown in Fig 3. The cellular target of the drug, the concentration used, and the cellular function(s) inhibited by each drug are indicated. B) Quantification of cytotoxicity for the drugs that inhibited SFV infection in Fig 3 in MEF DKO cells pre-treated for 6 days with vehicle (Ctrl) or 4-OH-TMX to deplete dynamin 1 and 2. Cell viability was measured by Cell-Titer Glow assay. The staurosposin derivative UCN-01, known to induce apoptosis, was used at a concentration of 20 mM as a positive control for toxicity. The concentration of all the other drygs is indicated in A. C) Schematic representation of the sequential immunostaining protocol. After fixation and before permeabilization viruses on the surface of cells are immunostained using antibodies against the spike protein (Ab 1) followed by secondary antibodies conjugated to a fluorophore (e.g., excitation 488nm, virus out). After a second fixation and permeabilization, the immunostaining is repeated (Ab2) but using secondary antibodies conjugated to a different fluorophore (e.g., excitation 647nm, virus in). D) Digital detection of viruses immunostained with one phluorofore before permeabilization (virus out) and with a second phluorofore after permeabilization (virus in) as described in C. Image analysis was performed using the Imaris software. (TIF) [file ppat.1012690.s004.tif]

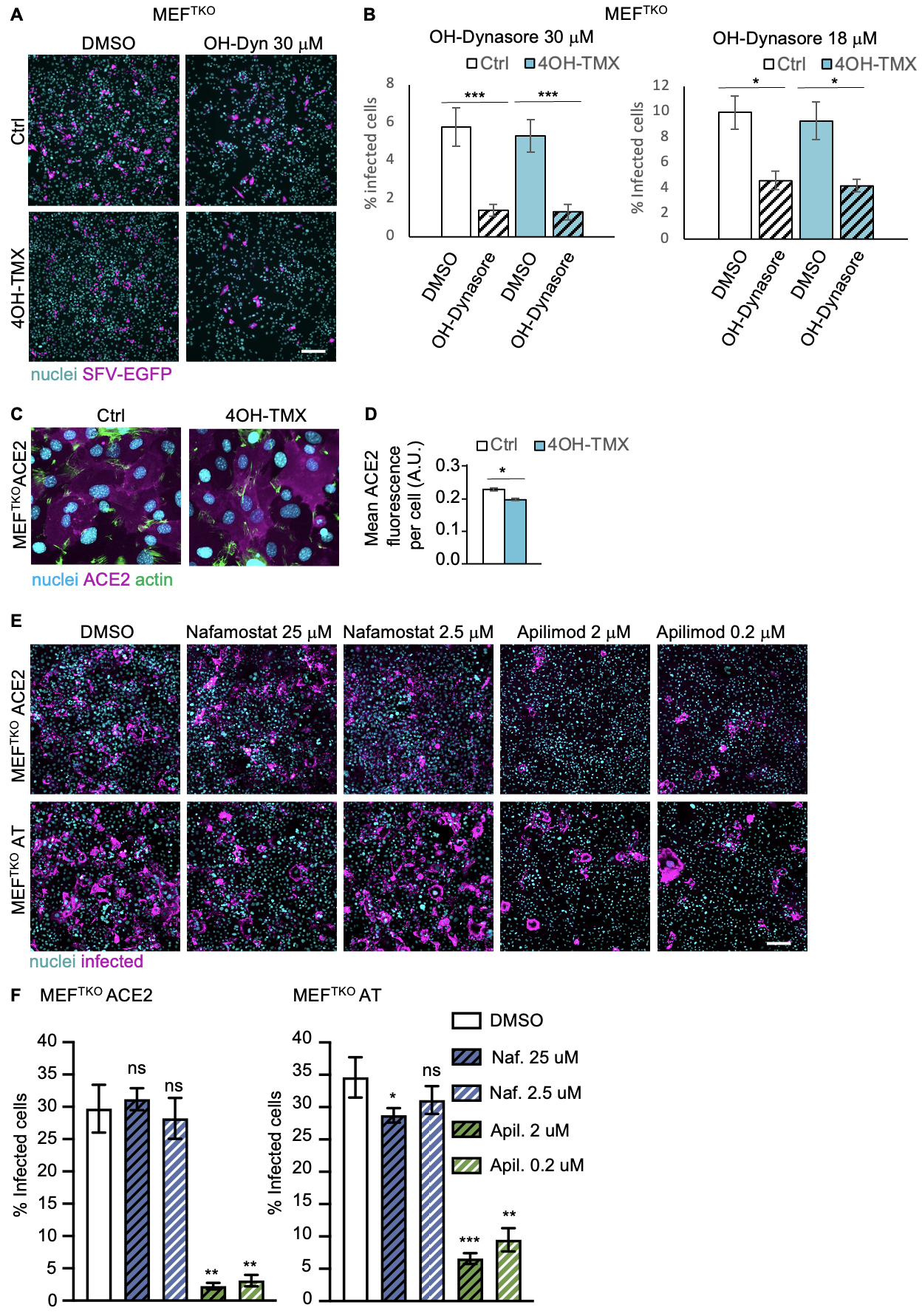

Supplement: S5 Fig — A) Representative fluorescence images of MEFTKO cells pretreated with vehicle control (Ctrl) or 4OH-TMX for 6 days and infected for 7 hours with SFV-EGFP (magenta) in the presence of 30 μM OH-dynasore (OH-dyn) or DMSO control. Nuclei are stained with Hoechst DNA die (cyan). Scale bar 100 μm. B) Quantification by automated image analysis of the experiment described in A with two concentrations of OH-dynasore, 30 and 18 μM,. Values represent the mean and standard deviation of four independent experiments. Data are normalized to the infection levels obtained in Ctrl cells treated with DMSO (indicated as 1). Statistical analysis was performed using ordinary two-way ANOVA multiple comparisons test (*p<0,05; *** p<0,001). C) Representative fluorescence images of MEFTKOACE2 cells 6 days after treatment with vehicle control (Ctrl) or 4OH-TMX. After fixation, non-permeabilized cells were processed for immunofluorescence detection of cell surface ACE2 (magenta) and actin fibers using phalloidin-A488 (green). Nuclei are stained with Hoechst DNA die (cyan). D) Quantification of the mean intensity of ACE2 fluorescent signal per cell suing automated image analysis. Values represent the mean of three replicas, each including more than 10.000 cells. A.U = arbitrary units. Statistical analysis was performed using an two tailed T-test (*p<0,05). E) Representative fluorescence images of MEFTKOACE2 and MEFTKOAT pretreated with nafamostat (25 μM or 2.5 μM), apilimod (2 μM or 0.2 μM) and infected with SARS-CoV-2 Wuhan. F) Quantification by image analysis of the experiment shown in E. Values indicate the mean of at least three independent experiments and the error bars represent STDEV. Statistical analysis was performed using ordinary two-way ANOVA multiple comparisons test (*p<0,05; ** p<0,01; ***p<0,001; n.s. = non-significant). (TIF) [file ppat.1012690.s005.tif]

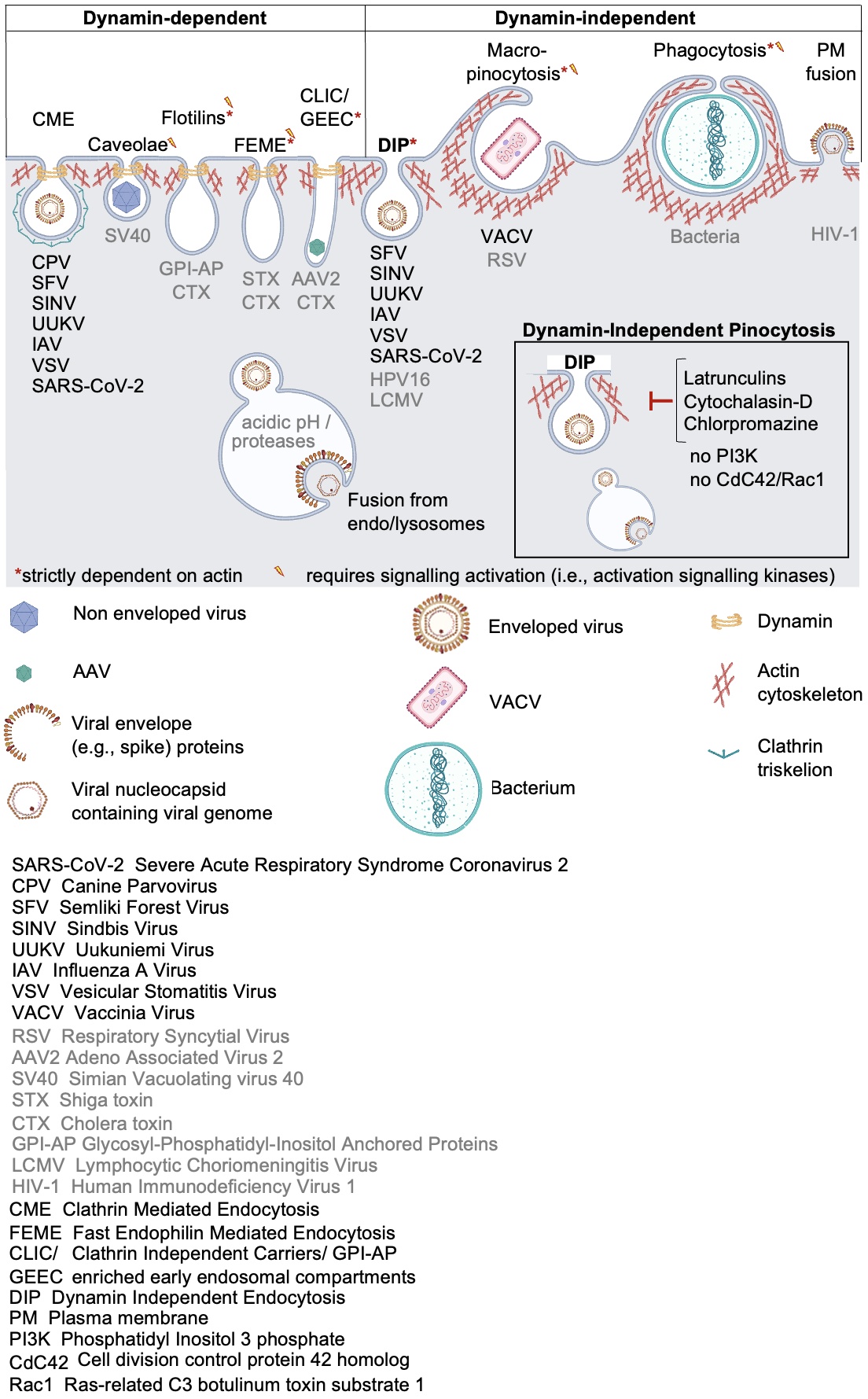

Supplement: S6 Fig — The viruses listed with black font were used in this study. The viruses and toxins in gray font were selected from literature. The inset shows inhibitors that bloked dynamin-independent virus infection and its independence from PI3K and Rho GTPases Cdc42 and Rac1, which makes this entry pathway mechanistically different from micropinocytosis. (TIF) [file ppat.1012690.s006.tif]
