# Supplementary material for: Dynamin independent endocytosis is an alternative cell entry mechanism for multiple animal viruses
Source: PLoS Pathog. 2024 Nov 14;20(11):e1012690. doi: 10.1371/journal.ppat.1012690 (PMC11594517; doi:10.1371/journal.ppat.1012690)
Supplement: Attachment — Submitted filename: 138164_1_rebuttal_2811618_s1yfsb.pdf [file ppat.1012690.s008.pdf]

Revision Plan

Manuscript number: RC-2023-02077
Corresponding author(s): Frederic Meunier, Merja Joensuu, and Giuseppe Balistreri

1. General Statements [optional]

The findings presented in this manuscript are original and have not been previously published, nor is the manuscript under consideration for publication by another journal. The authors of this manuscript declare to have no conflicts of interest.

1. Description of the planned revisions

We believe that incorporating the suggested corrections and conducting the additional experiments recommended by the reviewers will significantly enhance the quality of this study. These revisions will not only bolster the current conclusions but also broaden the relevance and applicability of our work to a wider scientific audience, extending beyond the field of virology.

As outlined in the following sections, we are fully committed to implementing the experiments proposed by the reviewers and making the necessary modifications to the manuscript in line with their suggestions. Our responses to each specific comment are provided in blue text.

Reviewer #1

Evidence, reproducibility and clarity

Summary: Several target cell entry pathways have been described for different viruses, including endocytic/ fusion pathways, some which are dynamin-dependent.

Here the authors exploited cell lines with multiple dynamin gene disruptions and other cell biological tools, as well as a phenotypic range of previously characterized viruses, to evaluate the relative importance of dynamin and actin for entry of viruses, including SARS-CoV-2.

In cells that lack the serine protease TMPRSS2, dynamin depletion blocked uptake and infection by SARS-CoV-2. Increasing the input virus partially rescued SARS-CoV-2 infection in the absence of dynamin, and both dynamin-dependent and dynamin-independent entry pathways were inhibited by drugs that disrupt actin polymerization.

Examination by electron microscopy indicated that the dynamin-independent endocytic process was clathrin-independent, in that, in the absence of dynamin, the majority of Semliki Forrest Virions were detected in bulb-shaped, non-coated pits. When TMPRSS2 was expressed, SARS-CoV-2 infection was rendered dynamin-independent.

Significance

Overall, the experiments are expertly performed, the results and conclusions are convincing, the text is clearly written and accurately describes the data, and the manuscript makes an important contribution to a complex and important topic in the cell biology of virus infection. It would be reasonable for the authors to publish the manuscript with the current data.

That being said, we have two main questions/comments:

1. The authors point out that SFV differs from SARS-CoV-2 in that it required actin only for the dynamin-independent entry. The EM experiments were done with SFV, not with SARS-CoV-2. This raises the question of the relevance for SARS-CoV-2 of the interesting finding that, in the absence of dynamin, SFV associated with non-coated pits.

If the authors had the tools to do similar EM experiments with SARS-CoV-2, it would be nice to include those results. Otherwise, it is fine to discuss/speculate about SARS-CoV-2 regarding this issue.
As requested by the reviewer, we are currently perform the suggested EM analysis of SARS-CoV-2 entry in the presence and absence of dynamins.

2. The authors show that TMPRSS2 allows the original Wuhan strain and Delta Variant of SARS-CoV-2 to bypass the need for dynamin. This is presumably because TMPRSS2 allows SARS-CoV-2 to fuse at the plasma membrane, precluding need for endocytosis altogether. The authors also mention literature claiming that Omicron is more dependent upon endocytosis than the Wuhan and Delta variants. If the authors had data with Omicron it would be really nice to include it.
We have already conducted this experiment and have incorporated the quantitative results into the updated version of the manuscript, now presented as Figure 8.

There were some minor typos/grammar/other quoted here:

- Ultrastructural analysis by electron microscopy showed that this dynamin-independent endocytic processes - cell injests particles and nutrients by encoulfing them - some viruses have been show
Thank you for noticing the error. We have modified the text as: "Ultrastructural analysis by electron microscopy showed that this dynamin-independent endocytic processes appeared as 150-200 nm non-coated invaginations that have been shown to be efficiently used by numerous mammalian viruses, including alphaviruses, influenza, vesicular stomatitis, bunya, adeno, vaccinia, and rhinovirus.".

- The final step of an endocytic vesicle formation culminates with the pinching of vesicle off from the PM into the cytoplasm
We have modified the sentence as: "The concluding stage of endocytic vesicle formation is marked by the vesicle being pinched off from the plasma membrane and released into the cytoplasm."

- For other viruses, such as respiratory viruses (This word is a little strange here since influenza was mentioned in the last sentence.)
Thank you for noticing the error, we have removed the mention to respiratory viruses: " For other viruses (including coronaviruses), the fusion is triggered by proteolytic cleavage of the spike proteins that, once cleaved, undergo conformational changes leading first to the insertion of the viral spike into the host membrane and, upon retraction, the fusion of viral and cellular membranes9,10.".

- Viruses that use a receptor that is internalized by dynamin-dependent endocytosis (e.g. CPV and the TfR) (just reminding that TfR is not a virus)
We have amended the sentence to avoid misunderstandings: "Viruses (e.g. CPV) that use a receptor (e.g. TfR) that are internalized by dynamin-dependent endocytosis cannot efficiently infect cells in the absence of dynamins.".

- that appeared surrounded by an electron dense coated
We have corrected the typo: "In $MEF^{DNM1,2\ DKO}$ cells treated with vehicle control, TEM analysis revealed numerous viruses at the outer surface of the cells (Figure 4 A), as well as inside endocytic invaginations that were surrounded by an electron dense coat, consistent with the appearance of clathrin coated pits[47,48] (CCP) (Figure 4 B)."

- The main virial receptor could be internalized using two endocytic
We have corrected the typo: "The main viral receptor could be internalized using two endocytic mechanisms, one mainly available in unperturbed cells (e.g. dynamin-dependent), the other activated upon dynamin depletion (i.e. dynamin independent)."

- Virus infection was determined by FACS analysis of virial induced EGFP

We have corrected the typo: 'Virus infection was determined by FACS analysis of EGFP (VAVC and VSV), mCherry (SINV) or after immunofluorescence of viral antigens using virus-specific antibodies (IAV X31 and UUKV).".

Reviewer #2

Evidence, reproducibility and clarity

Summary: Ohja et al. present an interesting study investigating dynamin independent endocytic entry mechanism of viral infection. Using a genetic KO of 2 dynamin isoforms they show impacts on the infection of a range of large and small DNA and RNA viruses.

They go onto show that SARS-CoV-2 may utilise a dynamin independent mechanism of infection that requires an intact actin cytoskeleton.

Significance

This work is of interest to the field of virology and has the potential to answer previously understudied entry mechanisms important for a wide range of viruses. It is a well presented piece of work overall.

Major Comments:

- The abstract does not in my opinion reflect the content of the paper and is too 'SARS-CoV-2' centric. The work involves the use of a range of viruses to first define a mechanism that is applicable to SARS-CoV-2 and I think the abstract and title should reflect this.

As per the reviewer's request, we will make revisions to the Title and Abstract. As a 'non SARS-CoV-2-centric' title we have amended the title to: Multiple animal viruses, including SARS-CoV-2, can infect cells using alternative entry mechanisms.

- In figure 1H the authors postulate that the reduced impact of dyn1,2 KO on SFV infection may be due to the interaction with heparin sulphate proteoglycans. Have the authors considered performing experiments using Heparin to block infection in their KO cells -/+ tamoxifen treatment?

As per the reviewer's request, we will perform the proposed heparin experiments for SFV.

- In Figure 2 the authors assess infection of a range of viruses in dyn1,2 KO cells showing differential effects in some viruses but not all.

Have the authors confirmed whether tamoxifen treatment and the subsequent KD of dyn1,2 effect surface expression of the entry receptors for the viruses tested?

Although in general blocking receptor endocytosis results in an increase in its cell surface levels, we agree with the Reviewer that the effect of dynamin depletion on receptors levels should be monitored at least for some of the viruses. To address the question raised by the reviewer, we will monitor the surface expression of SFV receptors VLDLR and ApoER2, and of the CPV receptor TfR in the presence and absence of dynamins.

We have already confirmed that there are no changes in the surface expression of SARS-CoV-2 receptor ACE2 in the absence of dynamin and this new data will be added to Figure 7.

- Additionally in this setting, dyn1,2 KD may impact on post entry steps in the virus life cycle such as the initial establishment of viral replication.

Can the authors either provide evidence as to how they have delineated measurement entry over replication or support their findings with psuedotyped virus-like-particles?

This is an important point. As suggested by the reviewer, we will perform infection experiments in the presence or absence of Dynamins using VLPs pseudotyped with SFV and VSV spikes.

In addition, several of our experiments already indicate that upon dynamin depletion, the main block in virus infection is at the step of cell entry: 1) Upon DNM-depletion, the decrease in SARS-CoV-2 infection strongly correlates with a proportional block in spike (Figure 5) and virions (Figure 7) endocytosis; 2) exogenous expression of even low levels of the cell surface protease TMPRSS2 rescued SARS-CoV-2 infection in cells devoid of dynamins, indicating that merely by-passing endocytosis restores virus infection; 3) as shown in Figure 1 H for SFV, and in Figure 2 for multiple viruses, increasing the multiplicity of infection increases the number of infected cells, indicating that when virions access the dynamin-independent entry route, cells can be efficiently infected; 4) the infection of both negative strand (i.e. Uukuniemi virus, UUKV, Figure 2) and positive strand (i.e. human Rhino virus, HRVA1, Figure S3 D-E) RNA viruses, as well as DNA viruses (i.e. Vaccinia, Figure 2, and Adenovirus-5, Figure S3 B-C) are not affected by dynamin depletion, arguing against a general negative impact of dynamin depletion on cellular protein synthesis or other basic cell functions required for virus replication.

- Figure 3, given the unexpected results with the dynamin inhibitors, could this experiment be repeated with the dyn1-3 triple KD presented in figures 5-8?
As requested by the reviewer, we will repeat the main inhibitor experiments presented in Figure 3 for SFV also in DNM TKO cells.

- Statistical analysis of imaging data in figure 4 would help with the conclusions.
We have already performed the requested statistical analysis and modified Figure 4 accordingly.

- Additionally, the authors comment that in the KD cells the viruses were trapped in 'stalled CCPs'. What morphological changes determine this classification?
As previously reported by Ferguson et al. (Developmental Cell, 2009), who developed the conditional MEF DNM knock out cell models, all CCPs are stalled at 6 days post induction of dynamin depletion. When observed by electron microscopy, stalled CCPs are readily identified by the presence of elongated, membranous narrow neck structures that connects the vesicle to the plasma membrane. We have clarified this description in the manuscript text and indicated the morphological features typical for a ‘stalled’ clathrin coated pit in Figure 4 F (black asterisk and white arrowheads).

- Concerning the SARS-CoV-2 work presented in figures 6-8, the use of exogenous expression of the viral entry receptors ACE2 and TMPRSS2 is a concern.
While the reviewer appreciates that this is a necessary step to allow entry into their MEF-dyn1-3 KD cells, exogenous receptor expression can result in artificial entry of the virus.
To support their findings, can the authors perform experiments in either cell lines endogenously expressing ACE-2/TMPRSS2 such as Calu3 or Caco2 and KD dyn1-3 using transient siRNA?
This experiment poses a challenge due to the inherent difficulty of transfecting Caco2 and Calu3 cells and the potential difficulty of achieving a robust (>80%) simultaneous knockdown of all three dynamin isoforms. This is one of the reasons why we chose the conditional knock out approach. Nevertheless, we are committed to attempting this experiment.

This approach would also provide more evidence for the role of TMPRSS2 presented in SF5 as the limited expression of this protease limits the robustness of the conclusions one can draw from the data presented.
We appreciate the reviewer's observation, and to address this concern, we plan to not only perform siRNA knockdown of dynamins in cells with endogenous ACE2 and TMPRSS2 but also endeavor to elevate the expression levels of TMPRSS2 in our MEF DNM1,2,3 TKO ACE2 cells. It's worth noting,

however, that this task presents a unique challenge since expression of TMPRSS2, a trypsin-like cell surface protease, leads to cell detachment even when expressed at moderate levels.

Minor comments & typo:

- Introduction paragraph 1 engulfing
The sentence has been amended: “To gain access into the host cell's cytoplasm where viral protein synthesis and genome replication take place, most animal viruses hijack cell’s endocytic pathways1 by which the cell engulfs particles and nutrients into vesicular compartments. “.

- Pg 13 - typo in 'Figurre 6B'
The typo has been corrected.

2. Description of the revisions that have already been incorporated in the transferred manuscript

- Regarding the Reviewer 1 request on the use of Omicron variants, we have already conducted the requested experiments and have incorporated the quantitative results into the updated version of the manuscript, now presented as Figure 8.

- Regarding the Reviewer 2 request on the EM data, we have already performed the requested statistical analysis and modified Figure 4 accordingly. We have also clarified the EM descriptions in the manuscript text and indicated the morphological features typical for a ‘stalled’ clathrin coated pit in Figure 4 F (black asterisk and white arrowheads).

3. Description of analyses that authors prefer not to carry out

Please include a point-by-point response explaining why some of the requested data or additional analyses <u>might not be necessary or cannot be provided within the scope of a revision</u>. This can be due to time or resource limitations or in case of disagreement about the necessity of such additional data given the scope of the study. Please leave empty if not applicable.
