# Supplementary material for: Dynamin independent endocytosis is an alternative cell entry mechanism for multiple animal viruses
Source: PLoS Pathog. 2024 Nov 14;20(11):e1012690. doi: 10.1371/journal.ppat.1012690 (PMC11594517; doi:10.1371/journal.ppat.1012690)
Supplement: Attachment — Submitted filename: Response to Reviewers PLOS Pathogens.pdf [file ppat.1012690.s009.pdf]

Response to Reviewers

Manuscript number: RC-2023-02077
Corresponding author: Giuseppe Balistreri
Co-corresponding authors: Frederic Meunier, Merja Joensuu

Reviewer #1

Evidence, reproducibility and clarity
Summary: Several target cell entry pathways have been described for different viruses, including endocytic/ fusion pathways, some which are dynamin-dependent.
Here the authors exploited cell lines with multiple dynamin gene disruptions and other cell biological tools, as well as a phenotypic range of previously characterized viruses, to evaluate the relative importance of dynamin and actin for entry of viruses, including SARS-CoV-2.
In cells that lack the serine protease TMPRSS2, dynamin depletion blocked uptake and infection by SARS-CoV-2. Increasing the input virus partially rescued SARS-CoV-2 infection in the absence of dynamin, and both dynamin-dependent and dynamin-independent entry pathways were inhibited by drugs that disrupt actin polymerization.
Examination by electron microscopy indicated that the dynamin-independent endocytic process was clathrin-independent, in that, in the absence of dynamin, the majority of Semliki Forrest Virions were detected in bulb-shaped, non-coated pits. When TMPRSS2 was expressed, SARS-CoV-2 infection was rendered dynamin-independent.

Significance
Overall, the experiments are expertly performed, the results and conclusions are convincing, the text is clearly written and accurately describes the data, and the manuscript makes an important contribution to a complex and important topic in the cell biology of virus infection. It would be reasonable for the authors to publish the manuscript with the current data.

That being said, we have two main questions/comments:
1. The authors point out that SFV differs from SARS-CoV-2 in that it required actin only for the dynamin-independent entry. The EM experiments were done with SFV, not with SARS-CoV-2. This raises the question of the relevance for SARS-CoV-2 of the interesting finding that, in the absence of dynamin, SFV associated with non-coated pits. If the authors had the tools to do similar EM experiments with SARS-CoV-2, it would be nice to include those results. Otherwise, it is fine to discuss/speculate about SARS-CoV-2 regarding this issue.

We thank the Reviewer for the comment and have now performed a quantitative ultrastructural EM analysis of authentic SARS-CoV-2 Wuhan strain entry. These experiments were performed similarly as our earlier experiments on Semliki Forest Virus entry in MEF Dynamin 1,2,3 KO cells stably expressing hACE2. The results, shown in the new Figure 4, confirm that both viruses are found in both clathrin-coated and non-coated endocytic processes. However, in unperturbed control cells, proportionally more SARS-CoV-2 virions were found in small (coated and non-coated) endocytic processes compared to SFV. Coronaviruses are larger (100-120 nm in diameter including the spikes) than alphaviruses (60-70 nm in diameter). The fact that more SARS-CoV-2 viral particles are seen in endocytic process at 15 min post entry is consistent with slower internalization kinetics of SARS-CoV-2 compared to SFV. Similar results have been obtained for Vesicular Stomatitis Virus (Cureton et al., PLOS Pathogens, 2009), which is also larger (100-120 nm length) in size than SFV. Vesicular Stomatitis Virus has slightly slower entry kinetics than SFV and, like SARS-CoV-2, requires an intact actin cytoskeleton for optimal clathrin mediated endocytosis. We have added these considerations in the main text (Lines 457-472).

2. The authors show that TMPRSS2 allows the original Wuhan strain and Delta Variant of SARS-CoV-2 to bypass the need for dynamin. This is presumably because TMPRSS2 allows SARS-CoV-2 to fuse at the plasma membrane, precluding need for endocytosis altogether.

Response to Reviewers

The authors also mention literature claiming that Omicron is more dependent upon endocytosis than the Wuhan and Delta variants. If the authors had data with Omicron it would be really nice to include it.

We have now added the results of two Omicron variants, B1 and Xbb, to the study. As hinted by the reviewer, the results indeed confirm that the Omicron variants prefer endosomal entry rather than TMPRSS2 fusion at the cell surface. These new results are now included in the revised Figure 8 and discussed in the main results (Line 474-498) and discussion (Line 528-535).

There were some minor typos/grammar/other quoted here:

- Ultrastructural analysis by electron microscopy showed that this dynamin-independent endocytic processes - cell injests particles and nutrients by encoulfing them - some viruses have been show

We thank the Reviewer for noticing the error. This sentence was removed from the text.

- The final step of an endocytic vesicle formation culminates with the pinching of vesicle off from the PM into the cytoplasm

This error has been corrected. We have modified the sentence as follows: "The fission process requires energy typically provided by one of the three dynamin isoforms (dyn1-3), all of which have GTPase activity." (Line 82-83)

- For other viruses, such as respiratory viruses (This word is a little strange here since influenza was mentioned in the last sentence.)

We thank the Reviewer for noticing the imprecision. We have removed the mention to respiratory viruses and modified the text as follows: " For other enveloped viruses (including coronaviruses), the fusion is triggered by proteolytic cleavage of the spike proteins which results in conformational changes, the insertion of the viral spike into the host membrane and the fusion of viral and cellular membranes.". (Line 97-99)

- Viruses that use a receptor that is internalized by dynamin-dependent endocytosis (e.g. CPV and the TfR) (just reminding that TfR is not a virus)

We apologise if the sentence was not clear. We have now amended the sentence as follows: "Small viruses, e.g. CPV, that target a receptor (e.g. TfR) that is internalized exclusively via dynamin-dependent endocytic routes cannot efficiently infect cells in the absence of dynamins.". (Line 176-178)

- that appeared surrounded by an electron dense coated

We have corrected the typo: "endocytic invaginations that were surrounded by an electron dense coat" (Line 283-284)

- The main virial receptor could be internalized using two endocytic

We have corrected the sentence as follows: "This could suggest that dynamin depletion blocks internalization of the main viral receptor," (Line 513-514)

- Virus infection was determined by FACS analysis of virial induced EGFP

We have corrected the sentence as follows: 'Virus infection was determined by FACS analysis of EGFP". (Line 1139-1140)

Response to Reviewers

Reviewer #2

Evidence, reproducibility and clarity
Summary: Ohja et al. present an interesting study investigating dynamin independent endocytic entry mechanism of viral infection. Using a genetic KO of 2 dynamin isoforms they show impacts on the infection of a range of large and small DNA and RNA viruses. They go onto show that SARS-CoV-2 may utilise a dynamin independent mechanism of infection that requires an intact actin cytoskeleton.

Significance

This work is of interest to the field of virology and has the potential to answer previously understudied entry mechanisms important for a wide range of viruses. It is a well presented piece of work overall.

Major Comments:

- The abstract does not, in my opinion, reflect the content of the paper and is too 'SARS-CoV-2' centric. The work involves the use of a range of viruses to first define a mechanism that is applicable to SARS-CoV-2 and <u>I think the abstract and title should reflect this.</u>

We thank the Reviewer for this suggestion. As per the reviewer's request, we have changed the title and the abstract to better reflect the use of a range of different viruses that allowed us to define an alternative cell entry mechanism applicable to SARS-CoV-2 and it´s variants. The new title is: "**Dynamin independent endocytosis is an alternative cell entry mechanism for multiple animal viruses**".

- In figure 1H the authors postulate that the reduced impact of dyn1,2 KO on SFV infection may be due to the interaction with heparin sulphate proteoglycans. Have the authors considered performing experiments using Heparin to block infection in their KO cells -/+ tamoxifen treatment?

As per the Reviewer's request, we have performed this experiment, and the results confirm that heparin inhibits infection both in the presence and absence of dynamins. These data are now shown in the new Figure S2 B-C and mentioned in the main result session of the manuscript (Line 173-185)

- In Figure 2 the authors assess infection of a range of viruses in dyn1,2 KO cells showing differential effects in some viruses but not all.
Have the authors confirmed whether tamoxifen treatment and the subsequent KD of dyn1,2 effect surface expression of the entry receptors for the viruses tested?

Although, in general, blocking endocytosis results in an increase in the cell surface levels of a given receptor (e.g., the transferrin receptor), we agree with the Reviewer that the effect of dynamin depletion on receptors levels should be monitored for the viruses that were strongly inhibited by dynamin depletion, namely canine parvovirus and SARS-CoV-2. To address the question raised by the Reviewer, we have now confirmed that upon dynamin depletion, the cell surface binding of Tf increased significantly, indicating that the cell surface expression of the Tf receptor has not decreased but rather increased. These results are included in the new Figure S1 D-E.
In addition, we now show by immunofluorescence analysis in non-permeabilized cells that there are no changes in the surface expression of the SARS-CoV-2 receptor ACE2 in the absence of dynamins. These new data were added to Figure S5 C-D. For all the remaining viruses, the paper shows that depletion of dynamins partially inhibits infection only at low viral doses, while it did not inhibit infection upon increasing the multiplicity of infection.

Response to Reviewers

- Additionally in this setting, dyn1,2 KD may impact on post entry steps in the virus life cycle such as the initial establishment of viral replication.
Can the authors either provide evidence as to how they have delineated measurement entry over replication or support their findings with psuedotyped virus-like-particles?

We thank the reviewer for pointing out this possible caveat. Several of our experiments already indicate that upon dynamin depletion, the main block in virus infection is at the step of cell entry: 1) As shown for SFV in the new figure S2 B, in dynamin depleted cells the number of infected cells decreased but not the fluorescence brightness of the ZsGreen reporter protein expressed by the recombinant virus used. This argues against a defect in virus protein synthesis or early replication; 2) Upon DNM-depletion, the decrease in SARS-CoV-2 infection strongly correlates with a proportional block in spike (Figure 5) and virions (Figure 7) endocytosis; 3) Exogenous expression of the cell surface protease TMPRSS2 rescued SARS-CoV-2 infection in cells devoid of dynamins, indicating that merely by-passing endocytosis restores virus infection (new Figure 8); 4) As shown in Figure 1 H for SFV, and in Figure 2 for multiple viruses, increasing the multiplicity of infection increases the number of infected cells, indicating that when virions access the dynamin-independent entry route, cells can be efficiently infected; 5) The infection of both negative strand (i.e. Uukuniemi virus, UUKV, Figure 2) and positive strand (i.e. human Rhino virus, HRVA1, Figure S3 D-E) RNA viruses, as well as DNA viruses (i.e. Vaccinia, Figure 2, and Adenovirus-5, Figure S3 B-C) are not affected by dynamin depletion, arguing against a general negative impact of dynamin depletion on cellular protein synthesis or other basic cell functions required for virus replication.

- Figure 3, given the unexpected results with the dynamin inhibitors, could this experiment be repeated with the dyn1-3 triple KD presented in figures 5-8?

As requested by the reviewer, we have repeated the dynamin inhibitor experiments presented in Figure 3 for SFV also in DNM 1,2,3 TKO cells. The results confirm that even in the complete absence of all three dynamin isoforms, small molecule inhibitors of dynamin efficiently block SFV infection confirming clear off-target effects of these drugs. These new results are included in the Figure S5 A-B.

- Statistical analysis of imaging data in figure 4 would help with the conclusions.

We have performed the requested statistical analysis and modified Figure 4 accordingly. We have also included the statistical analysis in the new results of the SARS-CoV-2 electron microscopy analysis (Figure 7).

- Additionally, the authors comment that in the KD cells the viruses were trapped in 'stalled CCPs'. What morphological changes determine this classification?

As previously reported by Ferguson *et al.* (Developmental Cell, 2009), who developed the conditional MEF DNM knock out cell models, all CCPs are stalled at 6 days post induction of dynamin depletion. When observed by electron microscopy, stalled CCPs are readily identified by the presence of elongated, membranous narrow neck structures that connects the vesicle to the plasma membrane. We have clarified this description in the manuscript text and indicated the morphological features typical for a ‘stalled’ clathrin coated pit in Figure 4 F (white arrowheads). Similar elongated membranous structures are visible in the SARS-CoV-2 EM pales Figure 7 F.

- Provide more evidence for the role of TMPRSS2 presented in SF5 as the limited expression of this protease limits the robustness of the conclusions one can draw from the data presented.

RESPONSE: To better support our results, we have re-made the cell lines stably expressing ACE2 and TMPRSS2 and used them at low passage. The new results show that the expression of TMPRSS2 doubles the infection levels in cells depleted of dynamins, demonstrating that the

presence of this serine protease can, indeed, by-pass the need for virus endocytosis allowing infection presumably by direct fusion at the cell surface.

Minor comments & typo:

- Introduction paragraph 1 engulfing

The sentence has been amended as follows: "the cells engulf particles and nutrients". (Line 72)

- Pg 13 - typo in 'Figure 6B'

The typo has been corrected.

- Description of the revisions that have already been incorporated in the transferred manuscript.

  Described above.

- Description of analyses that authors could not to carry out.

  Reviewer 2: ***Concerning the SARS-CoV-2 work presented in figures 6-8, the use of exogenous expression of the viral entry receptors ACE2 and TMPRSS2 is a concern. While the reviewer appreciates that this is a necessary step to allow entry into their MEF-dyn1-3 KD cells, exogenous receptor expression can result in artificial entry of the virus.***
  To support their findings, can the authors perform experiments in either cell lines endogenously expressing ACE-2/TMPRSS2 such as Calu3 or Caco2 and KD dyn1-3 using transient siRNA?

  This experiment poses a challenge due to the inherent difficulty of transfecting Caco2 and Calu3 cells and the difficulty of achieving a robust (>80%) simultaneous knockdown of all three dynamin 1,2,3 isoforms. This is one of the reasons why we chose the conditional knock out approach. We attempted the experiment and reached only modest (<40%) KD of Dynamin 1 and 2, both in Caco2 and Calu3 cells. The results of the experiments were, therefore, inconclusive. We acknowledge that repeating the main experiments in a more 'physiological' model is important and we are working on solving the technical caveats. Nevertheless, the use of a dynamin independent alternative entry pathway was observed for multiple animal viruses used in our study without receptor overexpression (SFV, SINV, VSV, UUKV, Influenza). In addition, the over-expression of the TfR to allow CPV infection did not allow this virus to by-pass the need for dynamin dependent entry, indicating that receptor over-expression per se does not result in aberrant entry pathways. We also note that to limit the expression levels of the ACE2 receptor to a minimum in the stably expressing MEF cells, we used a very low MOI of the respective lentivirus expression vector (MOI=0.3), which ensures only one lentivirus integration per cell.
  Ultimately, the studies on entry pathways of SARS-CoV-2 virus will await validation in primary muco-ciliary respiratory epithelial models grown at the AIR-liquid interphase. We are starting to explore this model in our laboratory and will make use of it in the future studies of respiratory virus entry.
