# Supplementary material for: Dynamin independent endocytosis is an alternative cell entry mechanism for multiple animal viruses
Source: PLoS Pathog. 2024 Nov 14;20(11):e1012690. doi: 10.1371/journal.ppat.1012690 (PMC11594517; doi:10.1371/journal.ppat.1012690)
Supplement: Attachment — Submitted filename: Point to point response to reviewers 2 PloS Path.pdf [file ppat.1012690.s010.pdf]

Point-by-point response to Reviewer's comments received on December 1st, 2023, from the journal PLOS Pathogens.

Reviewer #1

Part-I summary
This study investigates the role of dynamin-dependent endocytosis in entry of a broad spectrum of enveloped and non-enveloped viruses. Taking advantage of availability of the conditional double- and triple-dynamin KO MEF cells, the authors compared the ability of diverse viruses to infect these and control cells. Most viruses tested were able to infect dynamin KO cells. Intriguingly, the utilization of a yet poorly characterized dynamin-independent pathway was more efficient at higher MOIs. Infection of dynamin KO cells was more reliant on actin dynamics. In particular, the authors found that SARS-CoV-2 entry into ACE2-expressing cells is heavily dependent on dynamin and actin and this dependence was modestly alleviated by expression of TMPRSS2 that cleaves the Spike protein. Spike cleavage at the S2' position renders it fusion-competent and may direct the virus entry through a dynamin-independent pathway or enable direct fusion with the plasma membrane. Overall, the results reported in this manuscript are original and of interest the readership of PLoS Pathogens. Most experiments appear to be expertly performed.

However, there are several major issues that reduced enthusiasm for this work. A general concern is that the limitations of the conditional KO approach employed are not properly acknowledged. While the conventional dynamin KD or inhibition strategies suffer from incomplete suppression of dynamin expression and off-target effects, the conditional KO of dynamins that is achieved over the course of 6 days is also likely to perturb the cell physiology and have unanticipated consequences for viral infection. No attempts are made to characterize these cells and assess possible changes in the expression of cognate cellular receptors for the viruses tested in this study.

Response: We thank the reviewer for acknowledging that our work is original and of interest for the readership of PLOS Pathogens. In the revised version of the manuscript we have now confirmed that the expression levels of transferrin receptor (new Figure S1 D-E) and ACE2 (new Figure S5 C-D) are not significantly decreased in dynamin depleted cells.

Part II – Major Issues: Key Experiments Required for Acceptance
1. Cell viability under different treatment conditions, including the relatively harsh treatment with actin depolymerizing drugs, is not measured. This is essential for a proper interpretation of inhibition of viral infection.

Response: Before addressing the specific comments, this reviewer should be informed that on December 4th, 2023, the PLOS Pathogens Publishing Editor Alice Musson sent us an e-mail instructing us to disregard the comments of this reviewer because our paper had already been reviewed by a different set of Reviewers. The Editor apologised for the mistake and gave us instructions to follow our original revisions plan. We, therefore, addressed experimentally the requests of the first set of reviewers, as per the journal's instructions. Upon resubmission of our revised paper, the journal sent the revised version not to the first set of reviewers, the comments of which we had addressed, but to the second set (which included this reviewer #1). We notified the journal of this big mistake, but they did not do anything about it and, to our astonishment, told us that in addition to the first two, we now must address the comments of the new reviewers from December 1st, and even a completely new one (referred to as reviewer #3). So, in total 5 reviewers, despite having had told us to disregard the new set of reviewers. An unprecedented degree of misconduct, we feel. The e-mail from PLOS Pathogens Publishing Editor Alice Musson is copied below:

"Dear Giuseppe,

Thank you for your email. I apologise for the additional round of review your submission has gone through, as this was not necessary.

Now that a decision has been rendered, we would be grateful if you could please revise your manuscript based on your submitted revision plan.

Kind regards,

Alice

PLOS I OPEN FOR DISCOVERY
Alice Musson | Publishing Editor | Journal Editorial Operations
plos.org | California (U.S.) corporation #C2354500, based in San Francisco

Case Number: 08261663
ref:!00DU00Ifis.!500PM02dKfH:ref"

Regarding the potential cytotoxic effect of drugs, we fully agree with the reviewer, this is a very important point. Although this request was not put forward by the first set of reviewers, and despite the instructions of PLOS Pathogens to disregards all the comments of the new reviewers received on December 1st, we have now performed a full cell viability test, performed with the Cell-Titre glow assay, in MEF cells with or without dynamins for the drugs that inhibited SFV infection. Unlike the positive control OH-staurosporine (drug name UCN-01), known to induce apoptosis, no significant cytotoxic effects were observed for any of the drug treatments under the experimental conditions used in Figure 3. The new cell viability results are now shown in Figure S4 B.
In addition to the cell viability assay, as shown in Figure 3A for Semliki Forest virus, to limit potential cytotoxic effects that long incubations with drugs could induce, cells were treated with each drug only for one 45 minutes (figure 3 A, red line DMSO/Drugs), followed by washes and incubation in growth media devoid of drugs and containing 20 mM NH4Cl to prevent further virus penetration from endosomes. The experiment shows that our short latrunculin treatment does not per se have any effect on cell viability as demonstrated by the fact that SFV infection in the MEF cells increases upon Latrunculin and Cytochalasin treatments.

2a. There is an utter lack of information on the MEF dynKO cells stably expressing ACE2 or both ACE2 and TMPRSS2. Without validation of these cells lines, the results related to SARS-CoV-2 infection are not interpretable.

Response: To better characterize these cells in the contest of SARS-CoV-2 infection, in the revised version of the manuscript we provide the following information:
1) In the absence of human ACE2, murine cells such as MEF cells are not infectable by SARS-CoV-2. This is well established and now mentioned in the main text line 372.
2) In the absence of human ACE2 expression the soluble trimeric spike of SARS-CoV-2 does not even bind MEF cells (Figure S2 A).
3) The human ACE2 protein is detectable readily detectable at the cell surface of our MEF-ACE2 cells and the cell surface expression levels for not change upon dynamin depletion (Figure S5 C-D).
4) In addition to these controls, the manuscript includes inhibitor studies using small molecule inhibitors of endosome maturation (apilimod) and TMPRSS2 (Nafamostat). The results of this experiments shown that our MEF ACE2 and ACE2+TMPRSS2 cells recapitulate the entry mechanisms shown to occur in any other known cell model system: in the absence of TMPRSS2, infection required delivery to late endosomes, in the presence of TMPRSS2, infection can occur without delivery to late endosomes.
5) In addition to these experiments, the entry of the soluble trimeric spike and authentic SARS-CoV-2 in the MEF-ACE cells was directedly quantified and characterized in the presence and absence of dynamins by immunofluorescence (Figure 6 A-C) and at the ultrastructural level by quantitative EM analysis (Figure 7).
The experiments provided above provide all the evidence that is required to interpret the results of the SARS-CoV-2 entry studies and confirm that in respect to virus entry and viral protein synthesis, our MEF cell models recapitulate the all the known steps of SARS-COV-2 life cycle.

2b. It is mentioned that TMPRSS2-GFP expressing cells were FACS-sorted and only cells the lowest TMPRSS2 expression (no results shown) were used for experiments owing to the lack of adherence of cells expressing higher levels of this enzyme. This raises concerns regarding cell viability, especially after treatment with small molecule inhibitors and the ability to express functional levels of TMPRSS2 in MEF dyn KO cells.

Response: Regarding the low levels of TMPRSS2 in our MEF cells, upon request of one of the first reviewers, we have made the transgenic cells again, now achieving higher levels of TMPRSS2 as demonstrated by the clear increase in infection in Dynamin-depleted cells expressing TMPRSS2 (Figure 8 D, DMSO blue bar) compared to cells that did not express the cell-surface protease (Figure 8 A, DMSO blue bar).

In addition, while the endosome maturation inhibitor Apilimod inhibited infection completely in dynamin depleted cells (Figure 8 A-C, Apilimod blue striped bars), the expression of TMPRSS2 rescued the infection up to 40% of the DMSO vehicle controls (Figure 8 D-F, Apilimod blue striped bars). The point of this experiments was to demonstrate that by merely expressing even low levels of a suitable cell surface protease (i.e. TMPRSS2), the block in virus entry and infection induced by the dynamin depletion can be significantly relived. These 'rescuing' results also demonstrate that in our dynamin depleted cells infection is blocked only at the level of virus entry and not at downstream steps of the virus life cycle such as translation or transcription of viral genes.
In regard to the concern of cell viability raised by the reviewers, our MEF-ACE2 and MEF-ACE2-TMPRSS2 grow and divide at the same rate of normal MEFs (now stated in materials and methods and in main text), and the drugs used in our paper did not have cytotoxic effect as shown in a thorough cell viability assay now included in Figure S4 B for MEF DKO.

3. Related to the previous point, in Fig. S5, SARS-CoV-2 infection of TMPRSS2 expressing cells is only marginally inhibited by the TMPRSS2 inhibitor nafamostat and remains quite sensitive to apilimod (inhibitor of endosome maturation). In addition, TMPRSS2 expression only slightly increased SARS-CoV-2 infection relative to ACE2-only expressing cells. This is highly unusual, given the reported robust effects of TMPRSS2 expression in other cell types. The expression and activity of TMPRSS2 in the constructed MEF dyn KO cells must be examined and reported.

Response: We thank the reviewer for this insightful comment. We do not consider highly unusual that expression of TMPRSS2 does not have a strong effect of virus infection. We are not aware of evidence in the available literature showing that the overexpression of TMPRSS2 in cathepsin-competent cell lines has a "robust" effect on virus infectivity within the first 20 hours of infection. At best there is a strong increase in the cytopathic effect of the virus (e.g. in VeroE6-TMRSS2), and the presence of TMPRSS2 prevents the loss of the furin-cleavage site upon multiple passages. In cells where endosomal entry is efficient, such as VeroE6 and A549-ACE2, the overexpression of TMPRSS2 at the plasma membrane does not result in a strong increase in the number of infected cells. However, we have previously shown that if both TMPRSS2- and endosome-mediated entry are inhibited, infection is synergistically blocked.
Therefore, even if the expression levels of TMPRSS2-GFP are not the highest, the protein is active, and results can be interpreted. Indeed, in the new experiment provided in Figure 8, the expression of TMPRSS2 doubles the infection in cells where the endosomal route is inhibited by apilimod.

Part III – Minor Issues: Editorial and Data Presentation Modifications

1. The increased use of a dynamin-independent entry pathways at higher MOI can be presented more clearly throughout the paper. Instead of asking the reader to visually estimate the ratio of infection in control and dynamin KO cells, the infection ratio can be plotted to reveal the relative contributions of the two pathways.

Response: We thank the reviewer for this suggestion. We, however, prefer to present the data as they are as, in our opinion, the graphs are sufficiently clear.

2. The existence of an additional, dynamin-independent macropinocytosis-like pathway has been described for the influenza virus (https://doi.org/10.1371/journal.ppat.1001329) – a study that should probably be referenced here.

Response: Done. The reference was added in Line 202

3. The results shown in Fig. 6 are not readily interpretable. One must "eyeball" the effects of dyn KO and actin depolymerization. To show the LatA effect on control and dyn KO cells, the respective ratios of infection values can be plotted. In some cases, a log scale can be useful. Importantly, it is unclear why infections do not scale linearly with MOI. Minor: The authors interpret the results of Fig. 6 as supporting the ability of SARS-CoV-2 to use dynamin-independent pathway. Given that cell surface proteases other than TMPRSS2 have been implicated in SARS-CoV-2 Spike cleavage, can the authors rule out the possibility of direct fusion with the PM of dyn KO cells.

Response: While acknowledging the reviewer's suggestion, we think that the results with the actin depolymerizing drug Lat-B are sufficiently clear considering that this drug strongly inhibits infection in the presence of dynamin and completely inhibits infection in the absence of dynamins. By showing the actual percentages of infected cells, which drop from more than 20% in DMSO treated cells to near zero in dynamin depleted cells treated with Lat-B (Figure 6 F), we

think the reader can better appreciate how essential the actin cytoskeleton is in the dynamin-independent pathway. If the reviewer thinks that our presentation is clear enough, we would, rather, prefer to leave the graphs as they are.
Regarding the linear scaling of the MOI in Figure 6H, we do not know why diluting virus infection by a factor of two does not scale linearly with the percentage of infected cells. Nevertheless, the point of this experiment was to show that although SARS-CoV-2 infection us mainly dynamin-dependent (in the absence of TMPRSS2), increasing the virus load does result is sizable infection. In regard to the possible fusion of virus et the plasma membrane in dynamin-depleted MEF-ACE2 cells, the results of further experiments shown in Figure 8 and Figure S5 exclude this possibility because in the absence of TMPRSS2 overexpression, SARS-COV-2 infection is completely blocked by inhibition of endosomal maturation with Apilimod (Figure 8A-C, apilimod blue striped bar), supporting a requirement for endocytosis and endosome maturation in MEF cells that do not express TMPRSS2.

4. Why rely on the use of apilimod alone to assess the contribution of endocytic entry, when specific inhibitors of cathepsin L and other cathepsins have been widely used to inhibit endocytic entry of SARS-CoV-2?

Response: In our study, Apilimod was chosen because the identity of the protease(s) that mediates infection from endo-lysosomes in MEF cells is not known. In addition, within the time frame of our experiments, apilimod was well tolerated by the cells. The drug stocks are also very stable upon storage at -20C, and the drug is active at low concentrations (0.2 μM), which was more cost effective compared to Cathepsin inhibitors that work at micromolar concentrations and, in our hands, tend to lose potency few months after storage at -20C (not shown).

5. It is unclear if "single receptor" viruses, like CPV, are indeed exclusively binding to one receptor (e.g., TfR). Most viruses appear to bind non-specifically to the cell surface molecules. This brings us to the enhanced use of dynamin-independent endocytosis by virus at higher MOIs. Unfortunately, the mechanism of such virus input-dependence of entry route is not addressed in this study.

Response: We thank the reviewer for pointing out the importance of entry receptors as opposed to attachment factors. The canine parvovirus cannot infect cells in the absence of the cognate transferrin receptor. We have now included this negative control in the new Figure S2 A-B.

In the revised version of the manuscript, we have addressed the possible mechanism of dependence of virus dose with dynamin independent entry in discussion session line 515-529.
We discussed at least two alternative scenarios. Either viruses use one receptor that can be internalized by two endocytic mechanisms (which is what happens for the Cholera toxin B and EGF receptors, for instance), or viruses can use more than one receptor, which open the possibility of using a different endocytic pathway. The fact that higher doses are needed to infect dynamin depleted cells for some viruses indicated that their main entry route is dynamin dependent and that perhaps dynamin may affect virus biding affinity/avidity by inducing receptor clustering at endocytic pits.

6. Fig. 2: The infections do not scale linearly with virus inputs throughout. I'd consider repeating the experiments using lower MOI.

Response: We respectful disagree with the reviewer and do not find repeating these experiments necessary. In Figure 2 we demonstrate that multiple viruses can infect cells even in the complete absence of dynamins, and they do so quite efficiently.

7. Fig. 3: Negative controls using viruses that, unlike SFV, are resistant to some of the experimental manipulations are lacking. Also, inhibition of SFV infection by EIPA is shown in Fig. 3 but not mentioned or interpreted in Results.

Response: Done. We have now repeated the drug test with Vaccinia virus which is resistant to the drugs that inhibit SFV and, conversely, sensitive to drugs known to block micropinocytosis (Figure 3 C-D).

We apologies for not having discussed the results with the inhibitor EIPA. These results are now included and discussed in the main text, line 271-275.

8. No error bars or statistical analysis shown in Fig. S3.

Response: The experiments in Figure S3 were performed two times independently and therefore no statistical analysis was performed. The results of the two biological repetitions are, however, very similar and indicate that Adenovirus-C5 and Rhinovirus-A1 are not inhibited by dynamin depletion, regardless of virus dose.

9. The super-resolution PALM results presented in Fig. S4 appear out of place, as the relationship between single actin molecule diffusion coefficients or foci observed by phalloidin staining and the function of actin in the context of endocytosis and virus entry are unclear. Unless the modest changes in single actin molecule mobility in dynamin KO cells and endocytic activity is established, I'd consider removing these results, as these do not add much to understanding of alternative viral entry pathways.

Response: We agree with the reviewer. These results have been removed from the paper.

10. Fig. 4: The preference of virus for NCPs in dynamin KO cells appears marginal. However, there is rather strong preference for LEPs in control cells, which is not commented on. How is this observation compatible with virus entry via clathrin- and dynamin-dependent endocytosis?

Response: We apologies with the reviewer if the name Large Endocytic Processes (LEP) was misleading. LEP structures represent mostly endosomes and, occasionally, cross-sections of the plasma membrane. Indeed, when virus endocytosis is blocked, the number of virions detected in endosomal structures decreased, while the proportion of virions at the cells surface increased (Figure 4). We have clarified this in the main text Line 291 and renamed the LEP as Large endocytic/endosomal processes.
We also notify this reviewer that upon request of one of the first set of reviewers (from Review Commons), in addition to SFV, we performed a new thorough quantitative EM analysis of SARS-CoV-2 entry (Figure 7). The results of the SFV and SARS-CoV-2 study are consistent, when dynamin dependent endocytosis is blocked, a much smaller fraction of the virus is found in intracellular vacuolar structures that have the features of endosomes (indicated as LEP). Both SFV and SARS-COV-2 are found in both clathrin-coated and non-coated invaginations of the plasma membrane. We also point out that upon dynamin depletion, a larger fraction of SARS-CoV-2 virions was found in clathrin coated pits that sometimes had long narrow necks. This is consistent with viruses being 'trapped' in endocytic processes that are stalled at the cell surface, a process that has been extensively characterized when the double and triple conditional dynamin KO cells were developed by the de Camilli's lab. In the absence of dynamins, CCP can form and the polymerization of actin around the exposed neck of the CCP pulls the invagination towards the cytoplasm, followed by actin disassembly and collapse back to the original position. This was studied both in live cells and by 3D electron tomography. Our results on virus entry are, therefore, consistent with the effect of dynamin depletion on pinocytosis.

11. Fig. 5: The experiments measuring uptake of the SARS-CoV-2 spike ectodomain lack controls assessing S binding to ACE2-negative cells and showing the specificity of this binding to ACE2-expressing cells (e.g., competition with RBDs).

Response: Done. We now added images and quantifications of spike binding to cells not expressing ACE2 (Figure 5 A-B). The results show that in the absence of ACE2 the trimeric soluble spike did not bind to cells unspecifically.
The further competition experiment proposed by the reviewers will not be performed as, in addition to the negative results described above, the specificity of binding between the spike protein of SARS-CoV-2 and the human receptor ACE2 is well established, and the correct folding, molecular structure, and *in vivo* use of the soluble trimeric spike used in this study have already been fully characterized (see references in the main text).

Reviewer #2
Part I – Summary
Ohja et al. report on the entry pathways used by multiple animal viruses and find that cell entry can be achieved both in the presence and absence of dynamin isoforms. The authors perform a multitude of experiments, but a cohesiveness of the presentation is lacking. While multiple

viruses are used in cell culture experiments involving transfection and inhibitory compounds, only one of them is subjected to ultrastructural analysis. This prevents the extrapolation of results to multiple virus families, which appears to be their intention. I believe that it is interesting that virus inoculum dose could impact whether virus uptake/entry occurs in a dynamin-dependent or dynamin-independent manner, and I believe that more experiments cantered around that aspect of the article would have resulted in a better presentation.

Response: This Reviewer should be informed that on December 4th, 2023, the PLOS Pathogens Publishing Editor Alice Musson sent us an e-mail instructing us to disregard the comments of this reviewer because our paper had already been reviewed by a different set of Reviewers. The Editor apologised for the mistake and gave us instructions to follow our original revisions plan. We, therefore, addressed experimentally the requests of the first set of reviewers, as per the journal's instructions. Upon resubmission of our revised paper, the journal sent the revised version not to the first set of reviewers, the comments of which we had addressed, but to the second set (which included this reviewer #2). Not surprisingly, this reviewer #2 did not recognize our point-to-point response letter and wondered if we had paraphrased his/her comments. We certainly did not. What happened is that the reviewer simply received a letter that was addressing comments from different reviewers. We notified the journal of this big mistake, but they did not do anything about it and, to our astonishment, told us that in addition to the first two, we now must address the comments of the new reviewers from December 1st, and even a completely new one (referred to as reviewer #3). So, in total 5 reviewers, despite having had told us to disregard the new set of reviewers. An unprecedented degree of misconduct, we feel. The e-mail from PLOS Pathogens Publishing Editor Alice Musson is copied below:

"Dear Giuseppe,

Thank you for your email. I apologise for the additional round of review your submission has gone through, as this was not necessary.

Now that a decision has been rendered, we would be grateful if you could please revise your manuscript based on your submitted revision plan.

Kind regards,

Alice

PLOS I OPEN FOR DISCOVERY
Alice Musson | Publishing Editor | Journal Editorial Operations
plos.org | California (U.S.) corporation #C2354500, based in San Francisco

Case Number: 08261663
ref:!00DU00Ifis.!500PM02dKfH:ref"

We, non the less, thank the reviewer for thoroughly examining our paper. In addition to Semliki forest virus, and as a request of the first set of reviewers from Review Commons, we have now added quantitative ultrastructural analysis of SARS-CoV-2 entry (Figure 7). The other viruses used in this paper served as controls to demonstrate that multiple virus families can enter cells using different endocytic pathways. We did not intend to characterize in detail the endocytic mechanisms for each virus. We instead focused more on two viruses, Semliki Forest virus (as a reference) and SARS-COV-2, the entry mechanism of which is far from being fully characterized.

Part II – Major Issues: Key Experiments Required for Acceptance
Major points:

1. This study implicated various methods to investigate the role of dynamin during entry of various viruses, but with just one type of non-human cell examined, MEF. MEF are not physiologically relevant to many viruses studied in this work, especially SARS-CoV-2. At a minimum, the authors should verify the key conclusions with a more relevant cell line / primary tissue / organoid. The dynamin requirement for the entry of a given virus is likely to vary among cell lines and tissue types. This type of work needs to include primary human tissue of relevance to the virus being studied and should involve the study of endogenous cellular factors that facilitate uptake/entry, rather than requiring overexpression. Concluding on how TMPRSS2 interferes with cell adhesion in MEF cells is a good example of how cell line choice is likely to

bias the outcome of experiment and is likely a distraction that is unrelated to SARS-CoV-2 biology in human beings.

Response: We agree with the reviewer that validating results obtained from cell lines with more physiological models is a crucial step in determining the relevant mechanism(s) of virus cell entry in specific target tissues. Currently, we are not aware of efficient methods to simultaneously and efficiently deplete all isoforms of dynamins in any other cells type than the MEF cells used in our study, including primary respiratory epithelial cells grown at air-liquid-interphase, the primary target of respiratory viruses such as SARS-CoV-2. While we are working hard in that direction, at present, our work represents one of the few (if not the only) study where a 'clean' genetic depletion - instead of small molecules and RNAi - was used to study how efficiently different viruses can utilize dynamin-independent entry mechanisms. We also would like to point out that the role of endocytosis in the SARS-CoV-2 infection of primary respiratory cells has never been directedly addressed to date. To our knowledge, available studies are limited to investigating the role of endosomal pH and endo/lysosomal proteases but not whether the virus requires endocytosis. Similarly, the fusion of viruses at the plasma membrane has been inferred by the ability of viruses to escape inhibitors of endosome acidification and endo/lysosomal proteases. Here we show that indeed, expression of TMPRSS2 bypasses the inhibitory effect caused by dynamin depletion. This is strong evidence, we believe, supporting the role of TMPRSS2 in allowing direct fusion from the cell surface.

In our study, we aimed at establishing if the complete block of dynamin-dependent endocytic entry, which is efficiently and notoriously used by multiple viruses to infect cells, would lead to complete block of infection or if dynamin-independent entry mechanisms could be used to allow productive infection. We have demonstrated that this question cannot be asked using dynamin inhibitors because they block infection even in the absence of dynamins.

Our work is, therefore, exploring the potential for dynamin-independent endocytosis to serve as an internalization mechanism for specific receptor-mediated virus entry. This question is relevant because the extent to which viruses such as SARS-CoV-2 require endocytosis in primary tissues has never been directedly addressed. The molecular machineries that regulate endocytic pathways are highly conserved in mammals. Having validated a model system to study the molecular mechanism of a poorly characterized virus endocytic pathway (i.e., dynamin-independent endocytosis) is, in our opinion, a very valuable asset to the scientific community, both in the field of cell biology and microbiology. Unlike any other virus entry study of which we are aware, where small molecule inhibitors or transient and incomplete RNAi-mediated depletion of dynamin-1 or -2 has been used to determine the dependence of a given virus infection to dynamin-mediated endocytosis, we used complete genetic depletion of all three dynamin isoforms. Thanks to this approach we can demonstrate that: i) dynamin inhibitors are clearly unspecific, they block endocytosis and virus infection in the absence of dynamins to the same extent as they do in cells that contain dynamins; ii) viruses that have evolved to use receptors that are strictly internalized by clathrin mediated endocytosis, such as the canine parvovirus and the associated transferrin receptor, cannot infect cells in the absence of dynamin; iii) On the contrary, viruses that have been shown to utilize as entry factors either broadly distributed glycans, such as heparan sulphate and sialic acid proteoglycans, as in the case of the Alphavirus Semliki forest virus or influenza virus, respectively, seem to be able to use dynamin-independent endocytosis with different efficiencies. Upon request of the first set of Reviewers, in our revised paper we experimentally demonstrated that the infection of SFV is equally inhibited by heparin in cells that either have or don't have dynamins, supporting the previously reported notion that laboratory strains of SFV use heparan sulphate proteoglycans as an entry factors for both endocytic pathways (new Figure S2 C-D).

2. At this stage in the coronavirus pandemic, it is important that much experimentation be centred on Omicron subvariants.
However, the findings produced by the authors here on Omicron entry are unlikely to reflect the nature of Omicron entry into physiologically relevant airway tissue.
The authors state that other groups have suggested that Omicron entry occurs in a TMPRSS2-independent, endocytic manner. However, the work in those studies was also mostly performed in irrelevant transformed cell lines.
In those respects, the findings reported here on Omicron are neither novel nor interesting. Some references that the authors have failed to cite are those indicating that matrix metalloproteinases can serve as Spike-activating proteases in host cells, especially Omicron lineages. In particular,

there is evidence that the entry mechanism of Omicron into primary nasal epithelia is not endocytic but is MMP-dependent (Shi et al. bioRxiv 2023).

Response: While acknowledging the reviewer for these comments, we respectful and strongly disagree with all of them. Before going into each of these statements, this reviewer should know that we performed the experiments with the Omicron variants upon specific request of the first set of reviewers, of which clearly this reviewer#2 is completely unaware, indicating that the journal PLOS pathogen has failed to convey this crucial information. It is difficult to understand how that can be possible considering the large amount of work that we put into the revision following the instructions of the journal. What is also difficult to comprehend is how could the journal ask reviewer#2 to evaluate a paper and then first tell the authors to disregard the reviewer#2 comments and then, five months later, request to address them again. This is extremely unprofessional and very unfair for all the authors but also for the reviewer#2.

Regarding the reviewer#2 specific comments:
Firstly, the work suggesting Omicron variants do not efficiently use TMPRSS2 was performed in cell lines, yes, but one the important conclusion of that work turned out to be correct, Omicron variants are insensitive to TMPRSS2. Cell lines, which the reviewers considered "irrelevant transformed cell lines" were used to identify ACE2 as the main SARS-CoV-2 receptor, and the role of TMPRSS2 has also been extensively studies in cell lines. The conclusion of the requirement for endocytosis might or might not correspond to what happens in primary tissues, but indeed that work pointed to a different protease than TMPRSS2, which is extremely interesting and, again, correct. With a different and independent set of experiments, particularly using a block in endocytosis, we have validated these results and confirmed that Omicron variants cannot use TMPRSS2. Independent validations are important in science and that is why the first set of reviewers asked for this experiment.

Secondly, the reviewer points to paper form 2023 showing that in primary human nasal epithelial cells grown at the air-liquid interphase, inhibitors of TMPRSS2 do not inhibit Omicron variants but pan inhibitors of MMP/ADAM proteases do. No experiments were performed to address whether the MMP/ADAM dependent infection occurs from the plasma membrane or from early/recycling endosomes. In fact, the authors of the indicated paper state in the discussion that their "results suggest that processing of Spike by metalloproteinases enables fusion at the plasma membrane <u>and/or in early or recycling endosomes".</u> We argue that direct fusion at the plasma membrane - an event never directedly demonstrated but rather inferred for SARS-CoV-2- particularly in polarized ciliated epithelial cells might not lead to productive infection due to the very dense actin cytoskeleton underneath the apical plasma membrane. Internalization into an early/recycling tubular compartment, on the other hand, could in principle be beneficial to deliver viral particles underneath the thick actin cortex. Both dynamin-dependent and independent endocytosis have been documented at the apical side of polarized epithelial cells. What is the role of endocytosis in SARS-COV-2 infection of respiratory polarized cells? If it is required, which endocytic mechanism is the virus using? Our paper provides a framework for studding the cellular factors involved dynamin-dependent and independent endocytosis. Validations into primary cells will have to be addressed to validate the our findings in future studies but at present we do not have yet a robust and non-toxic method to block endocytosis in ALI cells. We are currently working in this direction.

3. The title of the article is misleading. The use of the term "alternative" without referencing a canonical entry pathway is inappropriate. I think the authors are trying to state that these viruses use a variety of entry pathways (that does not mean "alternative"). While the use of different viruses in this study seems like an advantage, it complicates the message of the article and dilutes its overall significance. It has already been known for a long time that the viruses tested here use a variety of entry pathways into human cells. The authors need to hone their story to emphasize a point of importance that is both novel and significant.

Response: We agree with the reviewer. We have removed the word alternative from the title and suggest the new title: Dynamin-dependent and -independent endocytic entry pathways can be used by for multiple animal viruses with different efficiency.

Part III – Minor Issues: Editorial and Data Presentation Modifications
There are a number of typos and English language/grammar could be improved.

Response: We apologies for the typos. In the revised manuscript e have done our best to correct typos and English language/grammar imprecisions.

Point-by-point response to Reviewer's comments received on May 5st, 2024 from the journal PLOS Pathogens.

Reviewer #2
Part I – Summary
The authors have revised their submission, but an accurate point-by-point response to the reviewers' comments is missing. It is unclear if this is accidental or deliberate, or if the authors were paraphrasing the reviewers' comments in their point-by-point response.

Response: Addressed above.

Part II – Major Issues: Key Experiments Required for Acceptance
The authors should upload a new point-by-point response to reviewers that uses the exact words of the reviewers, as laid out in the PLOS Pathogens decision letter on December 1st, 2023.

Response: Addressed above. On December 4th, 2023, the journal PLOS Pathogens gave us instructions to disregard the reviewers' comments received on December 1st.

Reviewer #3
Part I – Summary
This study used the Dyn-KO-MEFs to examine the cell entry dependency on cellular dynamins by a group of large and small animal viruses. The authors concluded that single-receptor viruses are exclusively dependent on dynamin for cell entry, whereas multi-receptor viruses can enter cells via both dynamin-dependent and dynamin-independent endocytic pathways. The experiments were properly controlled. Interpretation of the data can be improved, as explained below.

Part II – Major Issues: Key Experiments Required for Acceptance
Virus entry has been studied quite extensively for the past decades. An account of the different endocytic pathways that are exploited by different viruses will help to understand the relevance of the subject investigated in this study. For example, which endocytic pathways are dynamin-dependent or dynamin-independent, and are used by which viruses. In this context, a model at the end of the manuscript to summarize the findings will also be helpful, also highlight why studying dynamin in the context of virus entry is important.

Response: We agree and thank the reviewer for this suggestion. We have now included a model to summarize our results and put them in the context of published work in the field of virus entry (Figure S6).

No evidence is provided on which viruses use multiple receptors. For example, will depleting the known receptor of a virus block the entry/infection? Without this type of data, the link between dynamin-independent cell entry and multiple receptor usage cannot be established. One argument can be that at high doses (such as MOI=10 or 100), virus particles can aggregate during the concentration process and can be engulfed by cells in a receptor-independent and dynamin-independent way.

Response: We thank the reviewer for these comments. To address the reviewer question, we have now added data indicating that in the absence of its single receptor canine parvovirus cannot infect the MEF cells. To support the use of multiple heparan-sulphate receptors for SFV, we performed competition experiments with heparin showing that SFV is blocked in both dynamin depleted and control cells, indicating that heparan-sulphate proteoglycans are the entry factors for SFV in both cells.

Most of the experiments measure viral protein expression >20 hours after infection, not virus entry per se, this caveat should be acknowledged.

Response: Done. Virus and spike entry has been directedly assessed in Figure 4, Figure 5, Figure 6 A-C, and Figure 7.

It is stated that (line 228) MEF(DKO) cells stop dividing, how relevant then are the findings made with these KO cells, particularly viral protein expression levels were measured as a readout of virus entry? In another word, in healthy primary cells, when dynamin function is transiently inhibited, how much of the observations in this study can be reproduced? Related, in lines 242-245, the authors stated that CEM and dynamin inhibitors inhibited viruses in both the wild type and Dyn-KO cells; in addition to the off-target possibility, can the authors titrate the drug concentrations to circumvent this possible effect? These inhibitors are widely used in many studies.

Response: Cells division had no impact on cellular translation as demonstrated by the fact that most of the viruses included in this study did infect the dynamin depleted cells. Also, expressing TMPTRSS2 rescued infection of SARS-CoV-2, further indicating that the fact that cells stop dividing is not confusing the interpretation of the entry results. In addition, we note that primary fully differentiated muco-ciliary epithelial cells also do not divide.

In regard to the off-target effect of dynamin inhibitors, we have now included an experiment using a sub-optimal dose of OH-dynasore and found that it inhibited SFV to the same extent in dynamin plus and minus cells (Figure S5 B).

Part III – Minor Issues: Editorial and Data Presentation Modifications
Some typos to correct:

line 40, "we studies"

Response: corrected.

line 86, ref is missing
Response: corrected.

line 300, "this ration"
Response: corrected.

line 303, "where in CCP"
Response: corrected.

line 461, "37 C"
Response: corrected.

line 522, "The function"
Response: corrected.

Point-by-point response to Reviewer's comments received on August 17th 2023 from the journal consortium Review Commons.

Manuscript number: RC-2023-02077

Reviewer #1

Evidence, reproducibility and clarity
Summary: Several target cell entry pathways have been described for different viruses, including endocytic/ fusion pathways, some which are dynamin-dependent.
Here the authors exploited cell lines with multiple dynamin gene disruptions and other cell biological tools, as well as a phenotypic range of previously characterized viruses, to evaluate the relative importance of dynamin and actin for entry of viruses, including SARS-CoV-2.
In cells that lack the serine protease TMPRSS2, dynamin depletion blocked uptake and infection by SARS-CoV-2. Increasing the input virus partially rescued SARS-CoV-2 infection in the absence of dynamin, and both dynamin-dependent and dynamin-independent entry pathways were inhibited by drugs that disrupt actin polymerization.

Examination by electron microscopy indicated that the dynamin-independent endocytic process was clathrin-independent, in that, in the absence of dynamin, the majority of Semliki Forrest Virions were detected in bulb-shaped, non-coated pits. When TMPRSS2 was expressed, SARS-CoV-2 infection was rendered dynamin-independent.

Significance
Overall, the experiments are expertly performed, the results and conclusions are convincing, the text is clearly written and accurately describes the data, and the manuscript makes an important contribution to a complex and important topic in the cell biology of virus infection. It would be reasonable for the authors to publish the manuscript with the current data.

That being said, we have two main questions/comments:
1. The authors point out that SFV differs from SARS-CoV-2 in that it required actin only for the dynamin-independent entry. The EM experiments were done with SFV, not with SARS-CoV-2. This raises the question of the relevance for SARS-CoV-2 of the interesting finding that, in the absence of dynamin, SFV associated with non-coated pits. If the authors had the tools to do similar EM experiments with SARS-CoV-2, it would be nice to include those results. Otherwise, it is fine to discuss/speculate about SARS-CoV-2 regarding this issue.

Response: We thank the Reviewer for the comment and have now performed a quantitative ultrastructural EM analysis of authentic SARS-CoV-2 Wuhan strain entry. These experiments were performed similarly as our earlier experiments on Semliki Forest Virus entry in MEF Dynamin 1,2,3 KO cells stably expressing hACE2. The results, shown in the new Figure 7, confirm that both viruses are found in both clathrin-coated and non-coated endocytic processes. However, in unperturbed control cells, proportionally more SARS-CoV-2 virions were found in small (coated and non-coated) endocytic processes compared to SFV. Coronaviruses are larger (100-120 nm in diameter including the spikes) than alphaviruses (60-70 nm in diameter). The fact that more SARS-CoV-2 viral particles are seen in endocytic process at 15 min post entry is consistent with slower internalization kinetics of SARS-CoV-2 compared to SFV. Similar results have been obtained for Vesicular Stomatitis Virus (Cureton et al., PLOS Pathogens, 2009), which is also larger (100-120 nm length) in size than SFV. Vesicular Stomatitis Virus has slightly slower entry kinetics than SFV and, like SARS-CoV-2, requires an intact actin cytoskeleton for optimal clathrin mediated endocytosis.

2. The authors show that TMPRSS2 allows the original Wuhan strain and Delta Variant of SARS-CoV-2 to bypass the need for dynamin. This is presumably because TMPRSS2 allows SARS-CoV-2 to fuse at the plasma membrane, precluding need for endocytosis altogether. The authors also mention literature claiming that Omicron is more dependent upon endocytosis than the Wuhan and Delta variants. If the authors had data with Omicron it would be really nice to include it.

Response: Done. We have now added the results of two Omicron variants, B1 and Xbb, to the study. As hinted by the reviewer, the results indeed confirm that the Omicron variants do not use efficiently TMPRSS2. These new results are now included in the revised Figure 8 and respective text.

There were some minor typos/grammar/other quoted here:

- Ultrastructural analysis by electron microscopy showed that this dynamin-independent endocytic processes - cell injests particles and nutrients by encoulfing them - some viruses have been show

Response: We thank the Reviewer for noticing the error. This sentence was removed from the text.

- The final step of an endocytic vesicle formation culminates with the pinching of vesicle off from the PM into the cytoplasm

Response: This error has been corrected. We have modified the sentence as follows: "The fission process requires energy typically provided by one of the three dynamin isoforms (dyn1-3), all of which have GTPase activity." (Line 82-83)

- For other viruses, such as respiratory viruses (This word is a little strange here since influenza was mentioned in the last sentence.)

Response: We thank the Reviewer for noticing the imprecision. We have removed the mention to respiratory viruses and modified the text as follows: " For other enveloped viruses (including coronaviruses), the fusion is triggered by proteolytic cleavage of the spike proteins which results in

conformational changes, the insertion of the viral spike into the host membrane and the fusion of viral and cellular membranes.". (Line 97-99)

- Viruses that use a receptor that is internalized by dynamin-dependent endocytosis (e.g. CPV and the TfR) (just reminding that TfR is not a virus)

Response: We apologise if the sentence was not clear. We have now amended the sentence as follows: "Small viruses, e.g. CPV, that target a receptor (e.g. TfR) that is internalized exclusively via dynamin-dependent endocytic routes cannot efficiently infect cells in the absence of dynamins.". (Line 180-182)

- that appeared surrounded by an electron dense coated

Response: We have corrected the typo: "endocytic invaginations that were surrounded by an electron dense coat" (Line 286-287)

- The main virial receptor could be internalized using two endocytic

Response: We have corrected the sentence as follows: "This could suggest that dynamin depletion blocks internalization of the main viral receptor," (Line 520-521)

- Virus infection was determined by FACS analysis of virial induced EGFP

Response: We have corrected the sentence as follows: 'the percentage of infected cells was determined by FACS analysis of virus-induced expression of EGFP". (Line 1119-1120)

Reviewer #2

Evidence, reproducibility and clarity
Summary: Ohja et al. present an interesting study investigating dynamin independent endocytic entry mechanism of viral infection. Using a genetic KO of 2 dynamin isoforms they show impacts on the infection of a range of large and small DNA and RNA viruses.
They go onto show that SARS-CoV-2 may utilise a dynamin independent mechanism of infection that requires an intact actin cytoskeleton.

Significance

This work is of interest to the field of virology and has the potential to answer previously understudied entry mechanisms important for a wide range of viruses. It is a well presented piece of work overall.

Major Comments:

- The abstract does not, in my opinion, reflect the content of the paper and is too 'SARS-CoV-2' centric. The work involves the use of a range of viruses to first define a mechanism that is applicable to SARS-CoV-2 and I think the abstract and title should reflect this.

Response: Done. We thank the Reviewer for this suggestion. As per the reviewer's request, we have changed the title and the abstract to better reflect the use of a range of different viruses that allowed us to define an alternative cell entry mechanism applicable to SARS-CoV-2 and it´s variants. The new title is: "Dynamin independent endocytosis is an alternative cell entry mechanism for multiple animal viruses".

- In figure 1H the authors postulate that the reduced impact of dyn1,2 KO on SFV infection may be due to the interaction with heparin sulphate proteoglycans. Have the authors considered performing experiments using Heparin to block infection in their KO cells -/+ tamoxifen treatment?

Response: Done. As per the Reviewer's request, we have performed this experiment, and the results confirm that heparin inhibits infection both in the presence and absence of dynamins. These data are now shown in the new Figure S2 B-C and mentioned in the respective main result session of the manuscript.

- In Figure 2 the authors assess infection of a range of viruses in dyn1,2 KO cells showing differential effects in some viruses but not all.
Have the authors confirmed whether tamoxifen treatment and the subsequent KD of dyn1,2 effect surface expression of the entry receptors for the viruses tested?

Response: Although, in general, blocking endocytosis results in an increase in the cell surface levels of a given receptor (e.g., the transferrin receptor), we agree with the Reviewer that the effect of dynamin depletion on receptors levels should be monitored for the viruses that were strongly inhibited by dynamin depletion, namely canine parvovirus and SARS-CoV-2. To address the question raised by the Reviewer, we have now confirmed that upon dynamin depletion, the cell surface binding of Tf increased significantly, indicating that the cell surface expression of the Tf receptor has not decreased but rather increased. These results are included in the new Figure S1 D-E.
In addition, we now show by immunofluorescence analysis in non-permeabilized cells that there are no changes in the surface expression of the SARS-CoV-2 receptor ACE2 in the absence of dynamins. These new data were added to Figure S5 C-D. For all the remaining viruses, the paper shows that depletion of dynamins partially inhibits infection only at low viral doses, while it did not inhibit infection upon increasing the multiplicity of infection.

- Additionally in this setting, dyn1,2 KD may impact on post entry steps in the virus life cycle such as the initial establishment of viral replication.
Can the authors either provide evidence as to how they have delineated measurement entry over replication or support their findings with psuedotyped virus-like-particles?

Response: We thank the reviewer for pointing out this possible caveat. Several of our experiments already indicate that upon dynamin depletion, the main block in virus infection is at the step of cell entry: 1) As shown for SFV in the new figure S2 B, in dynamin depleted cells the number of infected cells decreased but not the fluorescence brightness of the ZsGreen reporter protein expressed by the recombinant virus used. This argues against a defect in virus protein synthesis or early replication; 2) Upon DNM-depletion, the decrease in SARS-CoV-2 infection strongly correlates with a proportional block in spike (Figure 5) and virions (Figure 7) endocytosis; 3) Exogenous expression of the cell surface protease TMPRSS2 rescued SARS-CoV-2 infection in cells devoid of dynamins, indicating that merely by-passing endocytosis restores virus infection (new Figure 8); 4) As shown in Figure 1 H for SFV, and in Figure 2 for multiple viruses, increasing the multiplicity of infection increases the number of infected cells, indicating that when virions access the dynamin-independent entry route, cells can be efficiently infected; 5) The infection of both negative strand (i.e. Uukuniemi virus, UUKV, Figure 2) and positive strand (i.e. human Rhino virus, HRVA1, Figure S3 D-E) RNA viruses, as well as DNA viruses (i.e. Vaccinia, Figure 2, and Adenovirus-5, Figure S3 B-C) are not affected by dynamin depletion, arguing against a general negative impact of dynamin depletion on cellular protein synthesis or other basic cell functions required for virus replication.

- Figure 3, given the unexpected results with the dynamin inhibitors, could this experiment be repeated with the dyn1-3 triple KD presented in figures 5-8?

Response: Done. As requested by the reviewer, we have repeated the dynamin inhibitor experiments presented in Figure 3 for SFV also in DNM 1,2,3 TKO cells. The results confirm that even in the complete absence of all three dynamin isoforms, small molecule inhibitors of dynamin efficiently block SFV infection confirming clear off-target effects of these drugs. These new results are included in the Figure S5 A-B.

- Statistical analysis of imaging data in figure 4 would help with the conclusions.

Response: Done. We have performed the requested statistical analysis and modified Figure 4 accordingly. We have also included the statistical analysis in the new results of the SARS-CoV-2 electron microscopy analysis (Figure 7).

- Additionally, the authors comment that in the KD cells the viruses were trapped in 'stalled CCPs'. What morphological changes determine this classification?

Response: As previously reported in live-cell imaging studies by Ferguson et al. (Developmental Cell, 2009), who developed the conditional MEF DNM knock out cell models, all CCPs are stalled at 6 days post induction of dynamin depletion. When observed by electron microscopy, stalled CCPs are readily identified by the presence of elongated, membranous narrow neck structures that connects the vesicle to the plasma membrane. We have clarified this description in the manuscript

text and indicated the morphological features typical for a 'stalled' clathrin coated pit in Figure 4 F (white arrowheads). Similar elongated membranous structures are visible in the SARS-CoV-2 EM pales Figure 7 F.

- Provide more evidence for the role of TMPRSS2 presented in SF5 as the limited expression of this protease limits the robustness of the conclusions one can draw from the data presented.

Response: To better support our results, we have re-made the cell lines stably expressing ACE2 and TMPRSS2 and used them at low passage. The new results in Figure 8 show that the expression of TMPRSS2 doubles the infection levels in cells depleted of dynamins or in cells where endosome maturation is blocked by apilimod treatments, demonstrating that the presence of this serine protease can, indeed, by-pass the need for virus endocytosis allowing infection presumably by direct fusion at the cell surface.

Minor comments & typo:

- Introduction paragraph 1 engulfing

Response: The sentence has been amended as follows: "the cells engulf particles and nutrients". (Line 72)

- Pg 13 - typo in 'Figure 6B'

Response: The typo has been corrected.

- Description of the revisions that have already been incorporated in the transferred manuscript.

  Described above.

- Description of analyses that authors could not to carry out.

  Reviewer 2: Concerning the SARS-CoV-2 work presented in figures 6-8, the use of exogenous expression of the viral entry receptors ACE2 and TMPRSS2 is a concern.
  While the reviewer appreciates that this is a necessary step to allow entry into their MEF-dyn1-3 KD cells, exogenous receptor expression can result in artificial entry of the virus.
  To support their findings, can the authors perform experiments in either cell lines endogenously expressing ACE-2/TMPRSS2 such as Calu3 or Caco2 and KD dyn1-3 using transient siRNA?

  Response: This experiment poses a challenge due to the inherent difficulty of transfecting Caco2 and Calu3 cells and the difficulty of achieving a robust (>80%) simultaneous knockdown of all three dynamin 1,2,3 isoforms. This is one of the reasons why we chose the conditional knock out approach. We attempted the experiment and reached only modest (<40%) KD of Dynamin 1 and 2, both in Caco2 and Calu3 cells. The results of the experiments were, therefore, inconclusive. We acknowledge that repeating the main experiments in a more 'physiological' model is important and we are working on solving the technical caveats. Nevertheless, the use of a dynamin independent alternative entry pathway was observed for multiple animal viruses used in our study without receptor overexpression (SFV, SINV, VSV, UUKV, Influenza). In addition, the over-expression of the TfR to allow CPV infection did not allow this virus to by-pass the need for dynamin dependent entry, indicating that receptor over-expression per se does not result in aberrant entry pathways. We also note that to limit the expression levels of the ACE2 receptor to a minimum in the stably expressing MEF cells, we used a very low MOI of the respective lentivirus expression vector (MOI=0.3), which ensures only one lentivirus integration per cell.
  Ultimately, the studies on entry pathways of SARS-CoV-2 virus will await validation in primary muco-ciliary respiratory epithelial models grown at the AIR-liquid interphase. We are starting to explore this model in our laboratory and will make use of it in the future studies of respiratory virus entry.
