# Supplementary material for: Dynamin independent endocytosis is an alternative cell entry mechanism for multiple animal viruses
Source: PLoS Pathog. 2024 Nov 14;20(11):e1012690. doi: 10.1371/journal.ppat.1012690 (PMC11594517; doi:10.1371/journal.ppat.1012690)
Supplement: Attachment — Submitted filename: Point to Point response to Reviewer.pdf [file ppat.1012690.s011.pdf]

Point-by-point response to Reviewers comments received on August 27th 2024 from the journal PLOS Pathogens.

Part I - Summary
Please use this section to discuss strengths/weaknesses of study, novelty/significance, general execution and scholarship.

Reviewer #2: In this additional revision, Ohja et al. have responded to most of my concerns, and in some cases, have done so adequately. However, there remain several points that have not been addressed.

Reviewer #3: The authors made great efforts to address comments from the reviewers, and provided as much new data as possible as suggested. The manuscript has been significantly improved.

Part II – Major Issues: Key Experiments Required for Acceptance
Please use this section to detail the key new experiments or modifications of existing experiments that should be <u>absolutely</u> required to validate study conclusions.

Generally, there should be no more than 3 such required experiments or major modifications for a "Major Revision" recommendation. If more than 3 experiments are necessary to validate the study conclusions, then you are encouraged to recommend "Reject".

Reviewer #2: Major:

1. *In their rebuttal letter, the authors state that "the role of endocytosis in the SARS-CoV-2 infection of primary respiratory cells has never been directly addressed to date. To our knowledge, available studies are limited to investigating the role of endosomal pH and endolysosomal proteases but not whether the virus requires endocytosis. Similarly, the fusion of viruses at the plasma membrane has been inferred by the ability of viruses to escape inhibitors of endosome acidification and endolysosomal proteases." This is precisely why I brought up the recent publication Shi et al. Nat Comm 2024 in my peer review report. This study uses inhibitors to indicate the Omicron, in contrast to Delta and SARS-CoV-2 from 2020, enters primary nasal epithelia at the air-liquid interface in a TMPRSS2-independent manner, but also in a cathepsin-independent manner. This suggests that Omicron may enter primary human cells by undergoing fusion at a non-endolysosomal compartment. Supporting this, use of the endolysosomal acidification inhibitor (and inhibitor of endosomal maturation) Bafilomycin A was shown to inhibit Delta and SARS-CoV-2 from 2020, but not Omicron. This suggests that Omicron does not require functional endolysosomal machinery in order to enter primary human epithelial cells. Furthermore, combining MMP inhibitors with Bafilomycin A inhibited Omicron to the same extent as MMP inhibitors alone, suggesting that Omicron likely uses MMP proteases to trigger fusion*

at the cell surface or early/recycling endosomes close to the cell surface. The authors should discuss these findings, and they can state their limitations (inability to prove cell surface fusion versus early/recycling endosome fusion) while presenting their own data in its context.

Response: As suggested by the reviewer, in the newly revised version of our manuscript we have ackowledged that in human nasal epithelial cells *"Omicron likely uses MMP proteases to trigger fusion at the cell surface or early/recycling endosomes close to the cell surface"* (line 607-616). Following the reviewer suggestions we stated the limitation of the cited study *("inability to prove cell surface fusion versus early/recycling endosome fusion")* (Line 618-637) and presented our own data in the context of respiratory epithelia and also other cell types that have been shown to be positive for SARS-COV-2 in humans and where the need for cell surface proteases vs endocytosis has not yet been completelly clarified (e.g., we have recently shown that endosome maturation is required for SARS-COV-2 infection in human neurons naturally susceptible to infection) (Line 117-119, Line 599-601).

The authors provide evidence supporting that Omicron infection occurs in a TMPRSS2-independent manner, but this has already been demonstrated by multiple groups (including the Shi et al. Nat Comm 2024 paper mentioned above). Therefore, this is not a new or useful finding in and of itself. The authors should review the literature claiming that Omicron does, or does not, undergo fusion in endosomes in human cells.

Response: Before addressing the specific comment, we remind this Reviewer that repeating the main SARS-CoV-2 experiments with the Omicron variants was a request of one of the reviewers from Review Commons and that the journal PLOS Pathogens gave us instructions to address the requests and questions of all the reviewers, including Reviw Commons' reviewers.

As suggested by the reviewer, in the newly revised version of our manuscript we have cited the papers that claim Omicron variants does or does not undergo fusion in endosomes of human cells, particulalry respiratory cells grown at AIR-liquid interphase and *in vivo* (Line 607-616)

2. I stand by the statement that, if we really want to understand the many ways by which human viruses enter human cells, experiments must be performed with human cells (and ideally, primary tissue). Even if it represents a clean genetic background to address dynamin isoforms involved in virus entry, results obtained in MEF cells can't be used to report "novel findings" about how human viruses enter human cells. These results can't be used to infer insight into the biology and pathogenesis of human viruses that cause disease in humans. The authors need to discuss the limitations of their own findings at length and state that it is unclear to what extent their findings can be extrapolated to human cells/tissue.

Response. As suggested by the reviewer, in the newly revised version of the mannuscript we have enphasised that "it is unclear to what extent their findings can be extrapolated to human cells/tissue." (Line 629-637). We also point out that because endosome maturation (and therefore endocytosis) seems to be required for the infection of human primary neurons in cultures (Reference 33, 79) , our model sustem could be useful to dissect the molecular mechanisms of virus entry in cell tupes where endosytosis is required (Line 599-601), provided that validations into the respective primary human cell type are provided (as stated in Line 629-637).

3. I'm not convinced that the authors have properly excluded macropinocytosis as one of the dynamin-independent endocytosis mechanisms at play in their experiments. The fact that EIPA inhibits infection to a certain degree supports that fluid-phase, macropinocytotic entry is taking place. Also, wortmannin is not a suitable inhibitor of macropinocytosis, as it is a pan class I/class III PI3K and mTOR inhibitor. Caution: the authors use the term "micropinocytosis" in this manuscript instead of the proper term macropinocytosis.

Response: We thank the reviwere for pointing a discrepancy in our results. The amiloride inhibitor EIPA, often used as one of the standard inhibitors for bona-fide macro-pinocytosis, inhibted infection of Semliki forest virus in WT as well as dynamin-depleted cells. In the newly revised version of the maniscript we have addressed this issue experimentally. We performed direct measurements of SFV virion entry as well as quantitative measurements of virus acidification in dynamin-depleted cells (Line 284-324). As detailed in the new figure 3 E-H, and Figure S4 C-D) we found that EIPA did not affect SFV virion entry. However, at the concentrations used to effectlivelly block macropinocytosis (80 uM), EIPA is know to neutralize endosomal pH (Ref 58). This could explain the inhibition of SFV infection in wt and dyn-depleted cells. To determine if this drug prevented the internalized SFV virions from undergoing acidification, we performed immuno fluorescence assays using an antibody that recognized the acidified form of the virus envelope protein E1. Indeed, treatment with EIPA prevented virion acidification indicatig that the compound EIPA interfere, as reported by others (Ref 58), with endosome acidification (and not with not SFV entry). Thus, the results demostrate that SFV entry in dynamin depleted cells is not inhibited by EIPA.

The class I PI3Ks are required for macropinocitic activation, as well as for other proceses as pointed by the reviewer. In our experiments wortmannin did inhibit Vaccinia virus infection (Figure 3 B) but not SFV (Figure 3 A).

Reviewer #3:

1. Dynamin-independent endocytosis and thus dynamin-independent entry of IAV, LCMV, HPV and other viruses have been documented, as highlighted in the 2010 review article (Figure 1, ref 1), this needs to be well presented in the Introduction so that the

novelty and importance of this study can be justified, i.e. contributions to what is already known in the literature.

Response: As suggested by the reviewer, we have now referred to the known entry mechanisms proposed fto date or IAV, LCMV, HPV, and highlighted the contribution of our findings to what is already known in litterature (Line 86-95).

2. MEFs are not the natural target cells of many viruses that were investigated in the study, some of the findings thus may not be reproducible for a virus in its primary target cells, this weakness needs to be acknowledged.

Response. This issue was rised also by Reviewer#2. In the newly revised version of the manuscript we have acknowledged the limitation of our model system (Line 629-637).

3. Half of results centered on SARS-CoV-2, which can be shortened, some of the results are confirmative in nature, because similar findings have been reported in the literature, such as TMPRSS2-independent entry of the Omicron variant.

Response: We remind the reviewer that extending the SARS-CoV-2 work was a request of the first set of Reviewers from Review commons, which we had to address as per request from PLOS Pathoges (including quantitative strudies of SARS-CoV-2 entry by Electron Microscopy and all the work with the Omicron variants). We, therefore, cannot remove or shorten these results because they were specifically asked by other reviewers.

Part III – Minor Issues: Editorial and Data Presentation Modifications
Please use this section for editorial suggestions as well as relatively minor modifications of existing data that would enhance clarity.

Reviewer #2: N/A

Reviewer #3: (No Response)

PLOS authors have the option to publish the peer review history of their article (what does this mean?). If published, this will include your full peer review and any attached files.

If you choose "no", your identity will remain anonymous but your review may still be made public.

Do you want your identity to be public for this peer review? For information about this choice, including consent withdrawal, please see our Privacy Policy.

Reviewer #2: No

Reviewer #3: No

Figure Files:

While revising your submission, please upload your figure files to the Preflight Analysis and Conversion Engine (PACE) digital diagnostic tool, https://pacev2.apexcovantage.com. PACE helps ensure that figures meet PLOS requirements. To use PACE, you must first register as a user. Then, login and navigate to the UPLOAD tab, where you will find detailed instructions on how to use the tool. If you encounter any issues or have any questions when using PACE, please email us at figures@plos.org.

Data Requirements:

Please note that, as a condition of publication, PLOS' data policy requires that you make available all data used to draw the conclusions outlined in your manuscript. Data must be deposited in an appropriate repository, included within the body of the manuscript, or uploaded as supporting information. This includes all numerical values that were used to generate graphs, histograms etc.. For an example see here on PLOS Biology: http://www.plosbiology.org/article/info%3Adoi%2F10.1371%2Fjournal.pbio.1001908#s5.

Reproducibility:

To enhance the reproducibility of your results, we recommend that you deposit your laboratory protocols in protocols.io, where a protocol can be assigned its own identifier (DOI) such that it can be cited independently in the future. Additionally, PLOS ONE offers an option to publish peer-reviewed clinical study protocols. Read more information on sharing protocols at https://plos.org/protocols?utm_medium=editorial-email&utm_source=authorletters&utm_campaign=protocols
