# Supplementary material for: Dynamin independent endocytosis is an alternative cell entry mechanism for multiple animal viruses
Source: PLoS Pathog. 2024 Nov 14;20(11):e1012690. doi: 10.1371/journal.ppat.1012690 (PMC11594517; doi:10.1371/journal.ppat.1012690)
Supplement: Attachment — Submitted filename: Point to Point response to Reviewers.pdf [file ppat.1012690.s012.pdf]

Point-by-point response to Reviewers comments received on October 19th 2024 from the journal PLOS Pathogens.

Reviewer Comments (if any, and for reference):

Reviewer's Responses to Questions

Part I - Summary
Please use this section to discuss strengths/weaknesses of study, novelty/significance, general execution and scholarship.

Reviewer #2: (No Response)
Reviewer #3: My critiques have been addressed.

Part II – Major Issues: Key Experiments Required for Acceptance
Please use this section to detail the key new experiments or modifications of existing experiments that should be absolutely required to validate study conclusions.

Generally, there should be no more than 3 such required experiments or major modifications for a "Major Revision" recommendation. If more than 3 experiments are necessary to validate the study conclusions, then you are encouraged to recommend "Reject".

Reviewer #2: The authors have nearly adequately addressed my concerns. However, their is slight revision required to how the authors cite the Omicron literature with regards to endosome/plasma membrane fusion. At line 584 of the Conclusions section, the authors state "Recent evidence has shown that this variant favours either endosomal cathepsin in cell lines or membrane type-matryx (spelling error - matrix) metallo proteinases (MT-MMPs) mediated entry rather than TMPRSS2-mediated infection in human nasal epithelial cells grown at air-liquid interphase (ALI)."
In addition to reference 84 cited here (Chan et al. Science Advances), the authors should include citation for Shi et al. Nature Communications 2024 (PMID: 38291024) as discussed in previous round of review. Also, at line 593, Shi et al. should be cited alongside reference 84. The authors failed to describe how Shi et al. demonstrates that inhibition of endolysosomal trafficking by bafilomycin A1 does not interfere with Omicron BA.1 infection of primary nasal ALI, while it does block WA1 and Delta infection. Meanwhile, three different MMP inhibitors inhibit BA.1 in this primary tissue. These findings indicate that is unlikely that Omicron requires endosomal cathepsins for its entry. Rather, the evidence strongly supports the involvement of MMPs at or near the cell surface.

RESPONSE: Done. Reference to the Shi et al. Nat.Com. 2024 paper (now reference 85) was added in the discussion sessions lines 584, 587, 588, 590, and in the following paragraph in line 598.

As requested by the reviewer, to better describe the main findings and the biological significance of the Shi et al. paper, we added the following text to the discussions starting from line 584: "This recent work demonstrated that inhibition of endolysosomal acidification/trafficking by bafilomycin A1 does not interfere with Omicron BA.1 infection of primary nasal ALI cells, while it does block Wuhan and Delta variant infections[85]. Meanwhile, three different MMP inhibitors inhibit BA.1 in these primary cells[85]. These findings indicate that it is unlikely that Omicron requires endosomal cathepsins for its entry in the nasal epithelium. Rather, the evidence strongly supports the involvement of MMPs at or near the cell surface[85]."

Reviewer #2: Furthermore, there are two typos at lines 574-575: please correct to "particular" and "In addition."

RESPONSE: Both corrected.

Reviewer #3: No more major issues.

Part III – Minor Issues: Editorial and Data Presentation Modifications
Please use this section for editorial suggestions as well as relatively minor modifications of existing data that would enhance clarity.
Reviewer #2: N/A
Reviewer #3: (No Response)

PLOS authors have the option to publish the peer review history of their article (what does this mean?). If published, this will include your full peer review and any attached files.
RESPONSE: YES, we would like the entire review history of our article to be publicly available including the first review from Review Commons and all the further rounds of review and revisions from PLOS Pathogens. Thank you.

If you choose "no", your identity will remain anonymous but your review may still be made public.
RESPONSE: we chose YES.

Do you want your identity to be public for this peer review? For information about this choice, including consent withdrawal, please see our Privacy Policy.

Reviewer #2: No

Reviewer #3: No

Figure Files:

While revising your submission, please upload your figure files to the Preflight Analysis and Conversion Engine (PACE) digital diagnostic tool, https://pacev2.apexcovantage.com. PACE helps ensure that figures meet PLOS requirements. To use PACE, you must first register as a user. Then, login and navigate to the UPLOAD tab, where you will find detailed instructions on how to use the tool. If you encounter any issues or have any questions when using PACE, please email us at figures@plos.org.

Data Requirements:

Please note that, as a condition of publication, PLOS' data policy requires that you make available all data used to draw the conclusions outlined in your manuscript. Data must be deposited in an appropriate repository, included within the body of the manuscript, or uploaded as supporting information. This includes all numerical values that were used to generate graphs, histograms etc.. For an example see here on PLOS Biology: http://www.plosbiology.org/article/info%3Adoi%2F10.1371%2Fjournal.pbio.1001908#s5.

Reproducibility:

To enhance the reproducibility of your results, we recommend that you deposit your laboratory protocols in protocols.io, where a protocol can be assigned its own identifier (DOI) such that it can be cited independently in the future. Additionally, PLOS ONE offers an option to publish peer-reviewed clinical study protocols. Read more information on sharing protocols at https://plos.org/protocols?utm_medium=editorial-email&utm_source=authorletters&utm_campaign=protocols
